# Insight into the underlying molecular mechanism of dilated cardiomyopathy through integrative analysis of data mining, iTRAQ-PRM proteomics and bioinformatics

**DOI:** 10.1186/s12953-023-00214-9

**Published:** 2023-09-22

**Authors:** Hongli Xiong, Zhe Zheng, Congcong Zhao, Minzhu Zhao, Qi Wang, Peng Zhang, Yongguo Li, Ying Zhu, Shisheng Zhu, Jianbo Li

**Affiliations:** 1 Department of Forensic Medicine, Faculty of Basic Medical Sciences, Chongqing Medical University, Chongqing, 400016 China; 2 Department of Forensic Medicine, Henan University of Science and Technology, Luoyang, 471023 Henan China; 3 Department of Forensic Medicine, Hainan Medical University, Haikou, 571100 China; 4 Faculty of Basic Medical Sciences, Chongqing Medical and Pharmaceutical College, Chongqing, 401331 China

**Keywords:** Dilated cardiomyopathy, Molecular pathways, Gene network, Bioinformatics, Mechanisms

## Abstract

**Background:**

DCM is a common cardiomyopathy worldwide, which is characterized by ventricular dilatation and systolic dysfunction. DCM is one of the most widespread diseases contributing to sudden death and heart failure. However, our understanding of its molecular mechanisms is limited because of its etiology and underlying mechanisms. Hence, this study explored the underlying molecular mechanism of dilated cardiomyopathy through integrative analysis of data mining, iTRAQ-PRM proteomics and bioinformatics

**Methods:**

DCM target genes were downloaded from the public databases. Next, DCM was induced in 20 rats by 8 weeks doxorubicin treatment (2.5 mg/kg/week). We applied isobaric tags for a relative and absolute quantification (iTRAQ) coupled with proteomics approach to identify differentially expressed proteins (DEPs) in myocardial tissue. After association analysis of the DEPs and the key target genes, subsequent analyses, including functional annotation, pathway enrichment, validation, were performed.

**Results:**

Nine hundred thirty-five genes were identified as key target genes from public databases. Meanwhile, a total of 782 DEPs, including 348 up-regulated and 434 down-regulated proteins, were identified in our animal experiment. The functional annotation of these DEPs revealed complicated molecular mechanisms including TCA cycle, Oxidative phosphorylation, Cardiac muscle contraction. Moreover, the DEPs were analyzed for association with the key target genes screened in the public dataset. We further determined the importance of these three pathways.

**Conclusion:**

Our results demonstrate that TCA cycle, Oxidative phosphorylation, Cardiac muscle contraction played important roles in the detailed molecular mechanisms of DCM.

**Supplementary Information:**

The online version contains supplementary material available at 10.1186/s12953-023-00214-9.

## Introduction

Dilated cardiomyopathy (DCM) is a series of heterogeneous cardiomyopathy characterized by left ventricular or biventricular dilatation and systolic dysfunction, which is based on the exclusion of congenital heart disease, hypertensive heart disease, coronary heart disease and cardiac valvular disease [1]. WHO defines DCM as a serious heart disease in which abnormalities in the structure and function of myocardium can lead to a variety of morbidities and deaths [2]. According to epidemiological reported, the prevalence of DCM is as high as 1:250, and it has been placed on the rise in recent years [3]. In the past few years, DCM has become one of the leading causes of heart failure and sudden cardiac death, accounting for 30%-40% of all heart failure cases and leading to the need for heart transplants across the globe [4]. Additionally, DCM has a poor prognosis, with a 5-year survival rate of about 50% [5].

The cause of DCM can be dependent on both genetic and non-genetic factors [6]. According to the statistical analysis, about 20%-35% of DCM patients are caused by genetic factors, including mutations in genes such as LMNA,MYH7,MYH6 and TNNT2 [7, 8]. Whereas, the etiology of non-genetic DCM is relatively complex, mainly including drug, chemical toxin and infection and so on [9]. To date, the pathogenesis of DCM has not been fully clarified, but it has something to do mainly with changes in force transmission and force generation, metabolite changes, and abnormal ion channels [3, 10]. Because its exact pathogenesis has not been studied clearly, and the lack of human myocardial tissue for research, the ultimate outcome of DCM patients is mostly death. Therefore, sudden cardiac death caused by DCM remains an unresolved public health issue.

Currently, Echocardiography, blood biochemistry analysis, magnetic resonance imaging (MRI), and endocrinology biopsy are currently used in the assessment of DCM. Although traditional MRI and CT scans are commonly used in clinical practice to diagnose DCM, they cannot provide reliable information about the tissue structure and cannot identify its potential causes [9]. EMB is currently considered as the "gold standard” for the diagnosis of heart diseases, histological, immunohistochemical and molecular biological techniques can be used to analyze myocardial tissue obtained by EMB, but it is restricted in clinic because of its high risk, invasiveness and high cost [11]. Moreover, sudden cardiac death (SCD) caused by DCM is mainly diagnosed according to the diagnostic criteria of “Hudson”, that is, negative indicators were excluded and explicit criteria were met. Due to this, how to objectively diagnose DCM was the key challenge and difficulty of current clinical medicine and forensic pathology research.

Currently, scholars from domestic and foreign countries have conducted numerous studies to explore the causes of DCM in fields, and have revealed the role of certain genes in the occurrence and development of DCM disease. Camargo A et al. [12] established the DCM classification model by analyzing the public data sets, and finally identified the potential biomarkers and drug targets of DCM. In addition, Luo X et al. [13] used bioinformatics approaches to reveal the underlying molecular mechanisms and therapeutic targets of DCM (chip dataset GSE1156). However, the results of these data analysis have not been verified and further systematic research has to be performed. In recent years, proteomics based on mass spectrometry technology has become the preferred method for studying proteins in depth [14]. Proteomics that has been extensively used to understand biological processes and solve many biomedical problems, such as colorectal cancer and epilepsy and so on [15, 16]. The iTRAQ technology is one of the relatively and absolute quantitative high-throughput screening technologies that has been widely used in recent years, which has the advantages of high labeling efficiency, high sensitivity, wide-ranging applications, and the ability to compare the protein expression levels of up to 8 samples simultaneously [17].

**Fig. 1:**
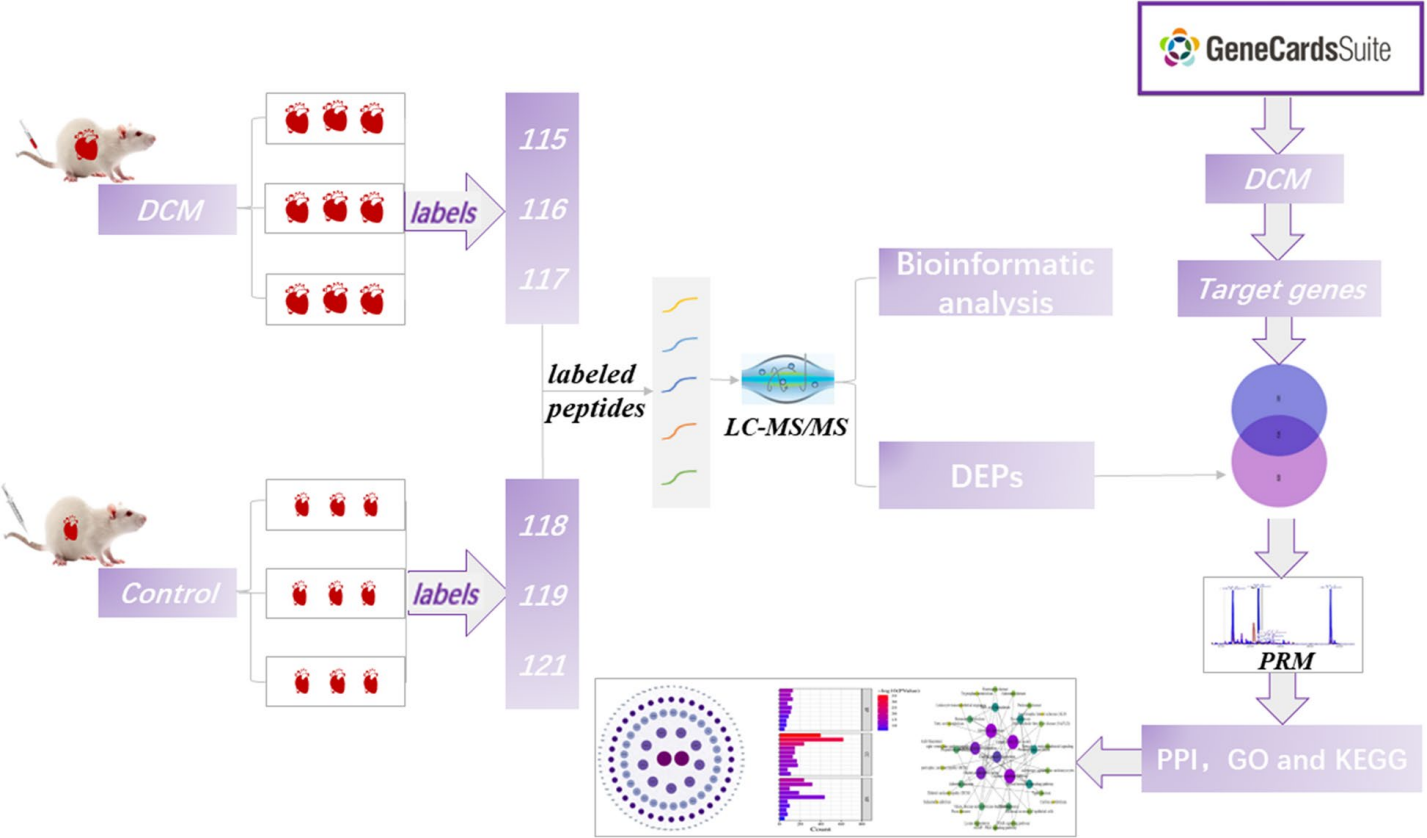
Flow diagram of experiment in this study

In this study, we established a rat model of DCM. Thereafter we elucidated the key proteins and pathways of DCM by integrating data mining, iTRAQ-PRM proteomics and bioinformatics analysis (Fig. 1). This study may provide new insights to understand the underlying molecular mechanism of DCM.

## Result

### Identification of key target genes

A total of 4066 target genes were downloaded from the database. Genes with relevance score ≥ 5.10 and protein coding were considered to be the key target genes of DCM. A total of 935 key target genes were identified for association analysis.

### Evaluation of the DCM rat model

#### Body weight changes and survival rates in rats

**Fig. 2:**
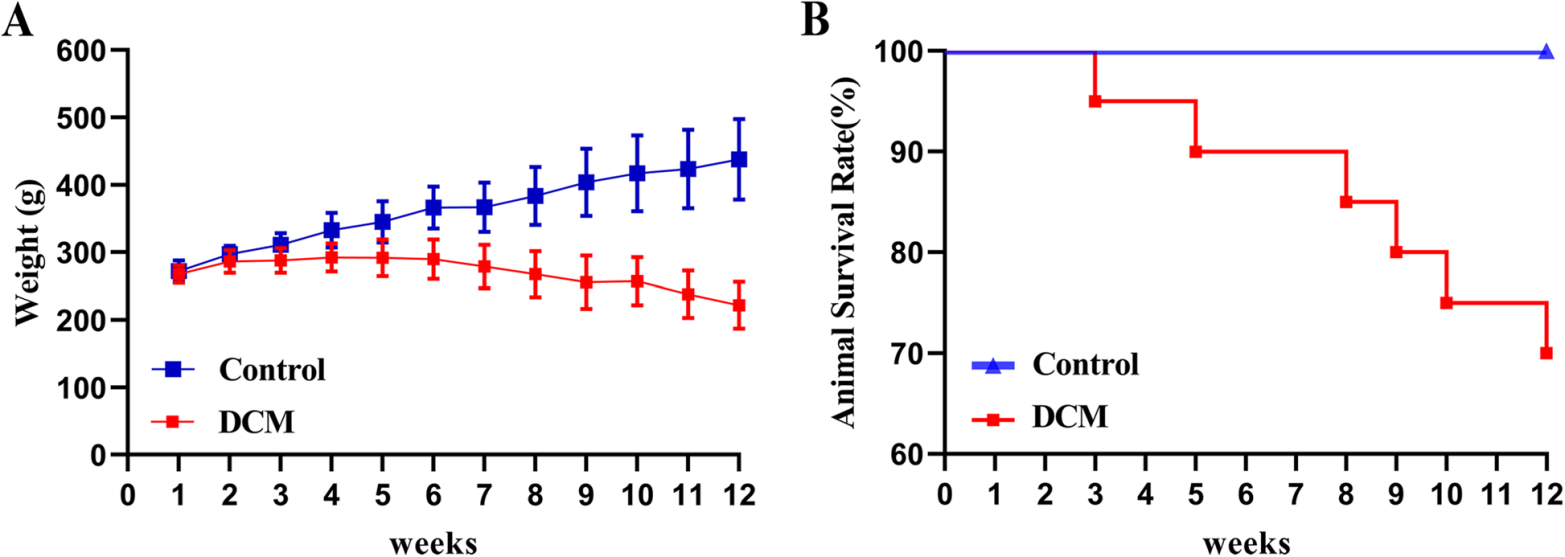
**A** The change trend of body weight of the two groups of rats. **B** Changes in the survival rate of the two groups of rats

Body weight of the DCM group was significantly reduced after the fourth week (Fig. 2A). We monitored the survival of the two groups of rats, and the results showed that a total of 6 rats died, with a survival rate of 70%, in the DCM group. No death was noted in the control group (Fig. 2B).

#### Echocardiography

**Fig. 3:**
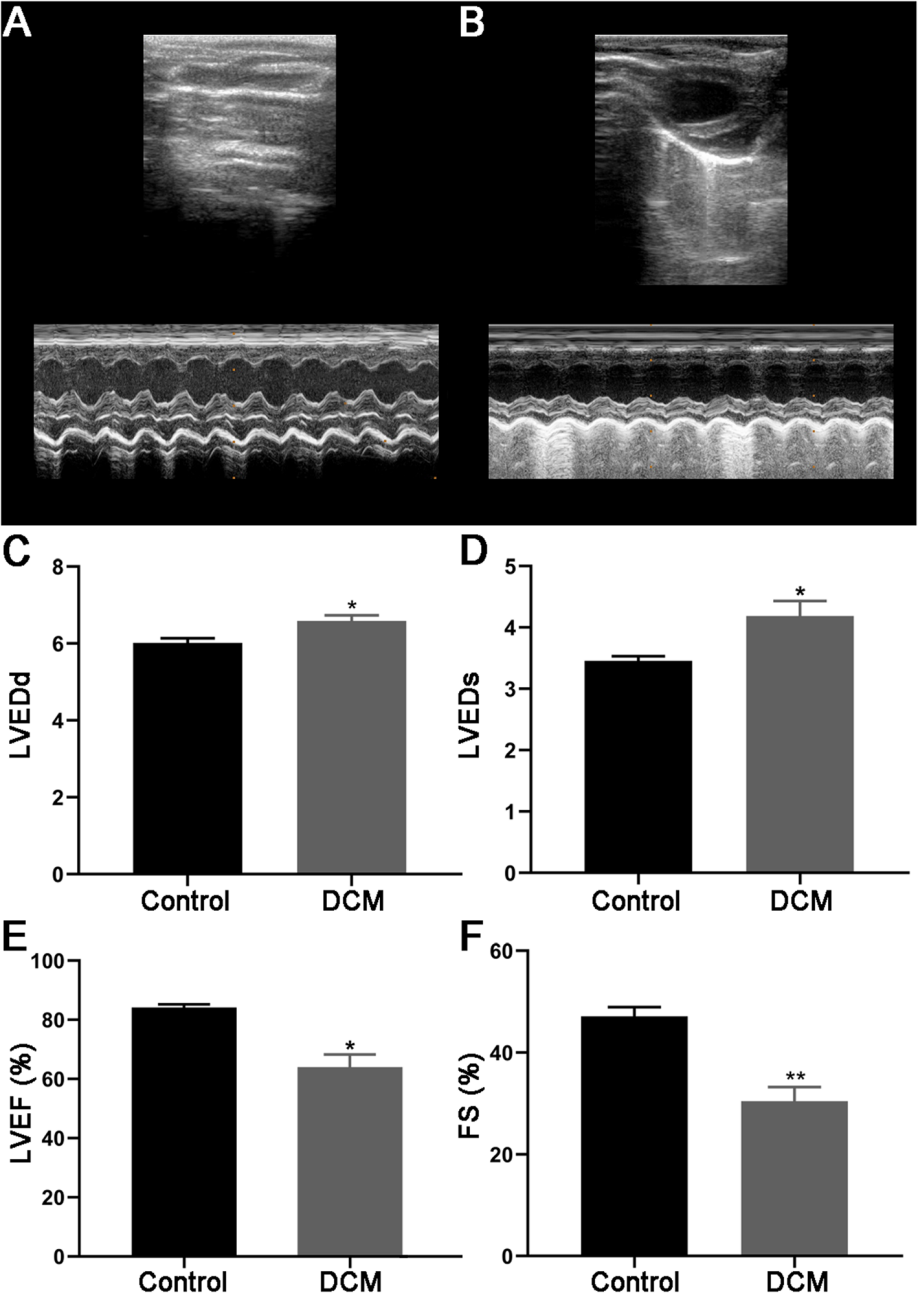
Representative echocardiographic images (**A** control; **B** DCM) and related cardiac function index of the two groups (**C** LVEDd, **D** LVEDs, **E** LVEF%, **F** FS%)

Cardiac function was evaluated by echocardiography, when compared with the control group, DCM group showed larger LV chamber dilatation in both diastole and systole (*P* < 0.0), suggesting that DCM rats had worse heart dilatation. However, DCM group had significantly reduced in LV systolic function as appraised by ejection fraction and fractional shortening (*P* < 0.01) when compared with control group (Fig. 3).

### Plasma pro-BNP

**Fig. 4:**
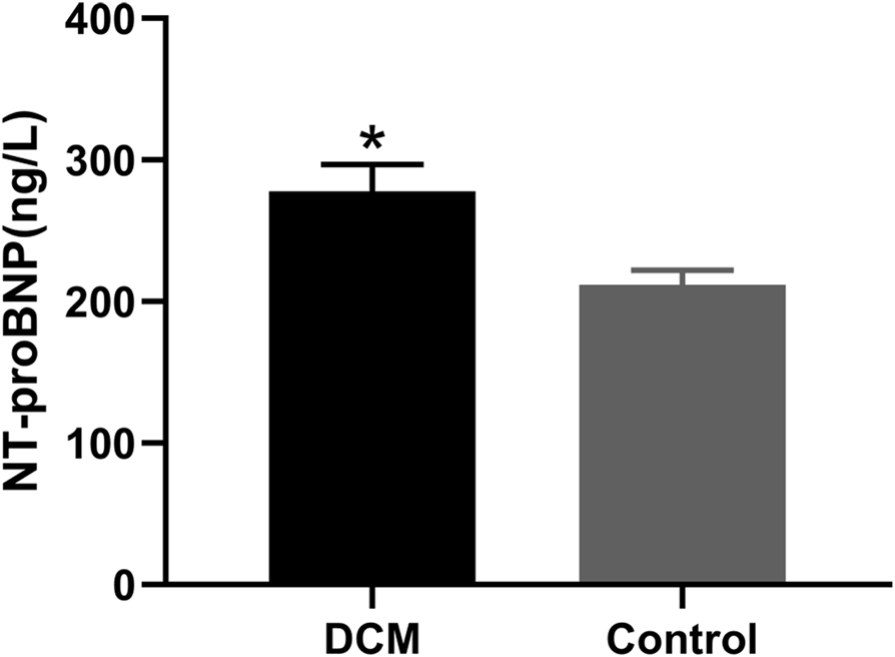
The changes of NT-proBNP levels in two groups of rats (* *p*<0.05)

It was consistent with the development of congestive heart failure that plasma pro-BNP levels were significantly higher in the DCM group than in the control group (Fig. 4).

### HE and Masson

**Fig. 5:**
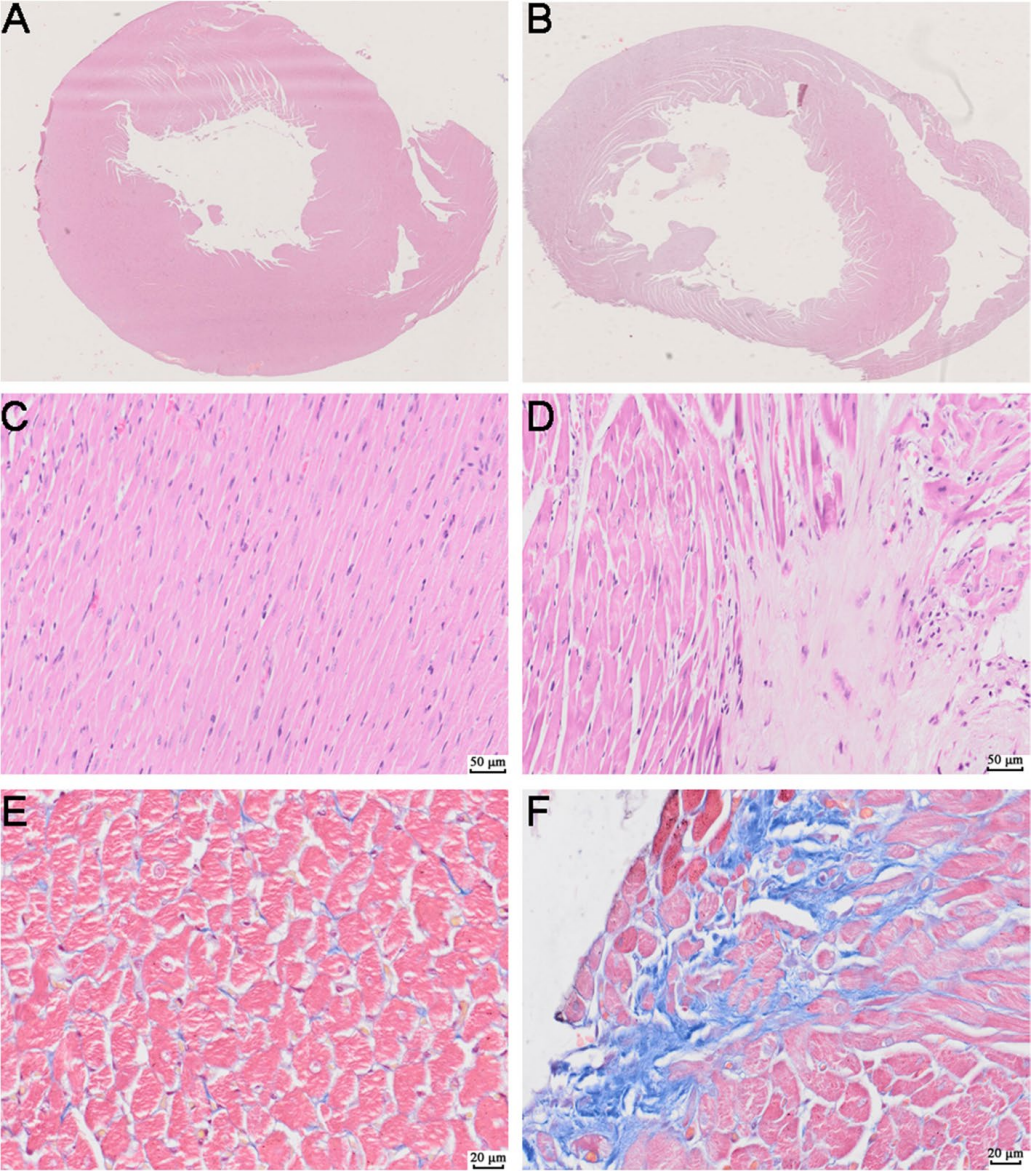
Histopathological changes of myocardium in two groups (**A**,** C**,** E** Control; **B**,** D**,** F** DCM). **A**,** B** cross section of myocardium. **C**,** D** Hematoxylin and eosin staining. **E**,** F** Masson trichrome stain

Autopsy study showed significantly enlarged heart size in DCM group. The left and right ventricles were dilated and the mural thrombosis was s in histological analysis of the cross-section of the heart stained with HE (Fig. 5A, B). In addition, HE staining revealed cardiomyocyte hypertrophy, disordered arrangement, interstitial loose edema, fibroblast proliferation, and myocardial interstitial fibrous connective tissue hyperplasia in DCM group (Fig. 5C, D). Meanwhile, DCM group also showed extensive fibrosis between bundles of myocytes as control group by Masson’s trichrome staining (Fig. 5E, F).

In conclusion, DCM animal models were successfully established, which is suitable for further iTRAQ-based proteomic analysis.

### Myocardial tissue proteomic analysis using iTRAQ

#### Identification of DEPs via iTRAQ coupled with LC–MS/MS

**Fig. 6:**
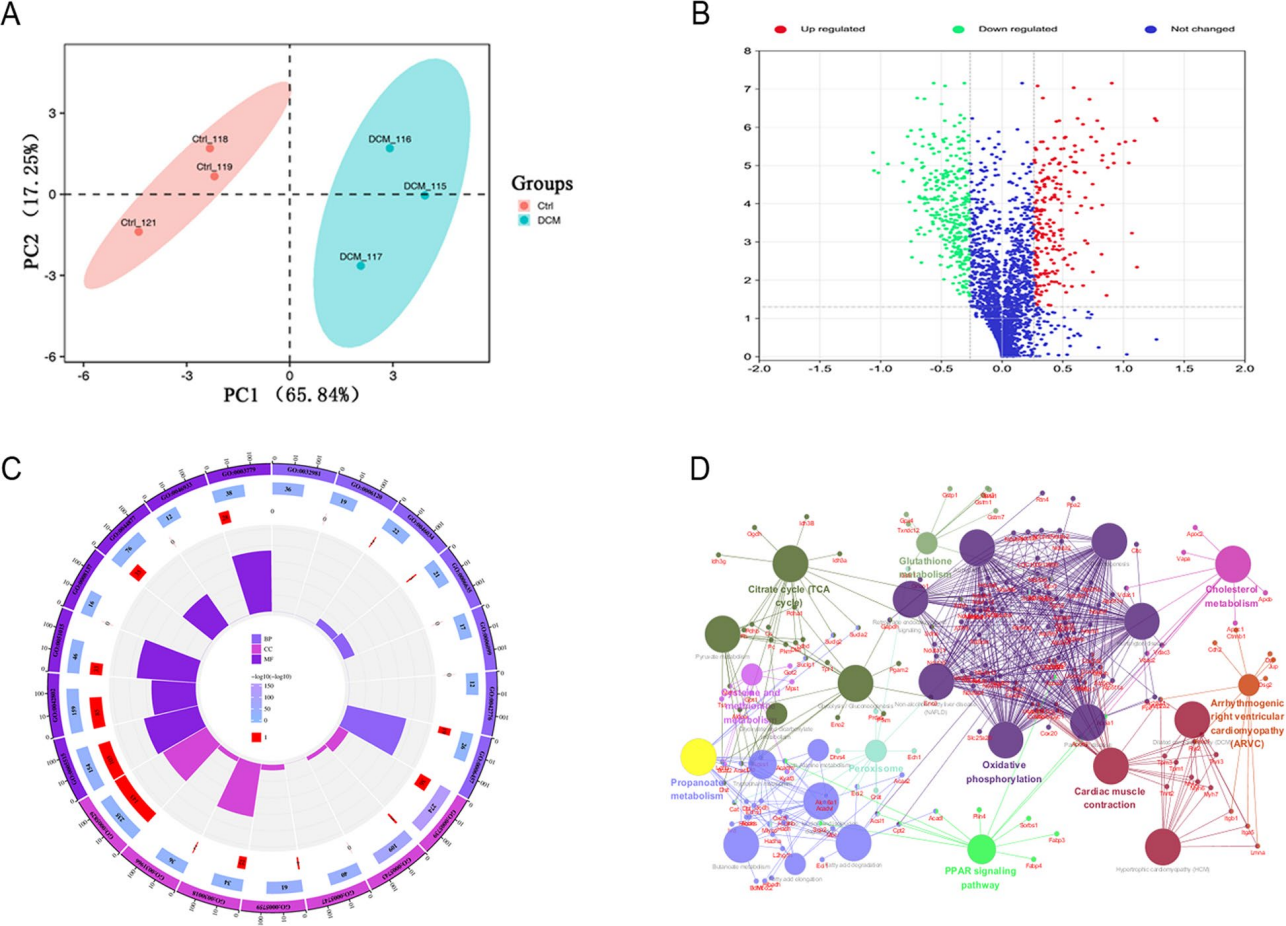
The functional annotation and enrichment analysis of the DEPs: (**A**,** B)** the principal component plot and volcano plot of protein changes in DCM and control groups. **C** The gene ontology (GO) analyses of DEPs. **D** KEGG enrichment pathways of DEPs

In order to capture global protein expression, the raw data of the iTRAQ were generated from the heart tissue samples of DCM and Control, with three biological replicates, respectively. The PCA results are presented in the Fig. 6A. A total of 2499 proteins were identified and quantified from all nine heart tissue samples, 1995 among which had at least two unique peptides (unique peptide numbers >1). In the myocardial tissues, proteins with expression ratios of over 1.5-fold increase or at least 0.67-fold decrease while adj *p*-value < 0.05 were considered to be differentially expressed, respectively [18]. As a result of the analysis, 782 DEPs were identified, including 348 proteins that were up-regulated and 434 proteins that were down-regulated (Fig. 6B, Table S1).

#### Functional categorization of DEPs

GO and KEGG functional classifications were performed to characterize the functions related to the identified DEPs. In terms of GO functional annotation, according to the standard correction *p*-value of 0.05, 180 significantly enriched GO terms were obtained, which were classified into three categories, including 94 biological processes (BP), 100 cellular component (CC), and 87 molecular functions (MF), respectively. For the 782 DEPs between the DCM and control comparison, these proteins were mainly concerned with the BP such as oxidation-reduction process, tricarboxylic acid cycle, 2-oxoglutarate metabolic process, protein folding. For the CC, these proteins were significantly enriched in mitochondrion, extracellular exosome, Z disc, mitochondrial inner membrane, focal adhesion and etc. Significant enrichment was observed in the MF for actin filament binding, NADH dehydrogenase activity, poly(A) RNA binding, protein complex binding, etc., (Fig. 6C).

A total of 49 significantly changed KEGG pathways were detected through KEGG pathway enrichment analysis (*p*-value < 0.05). Pathway analysis revealed that these proteins were involved in Oxidative phosphorylation, Carbon metabolism, Citrate cycle (TCA cycle), Cardiac muscle contraction, Parkin-son's disease, and other processes (Fig. 6D).

### Association analysis of key target genes and DEPs

To understand the specific mechanism of pathogenesis of DCM, we further performed an association analysis of target genes and DEPs. A total of 150 overlapping proteins were identified as key proteins in DCM (Table S2).

### PRM validation of the key DEPs in DCM

**Fig. 7:**
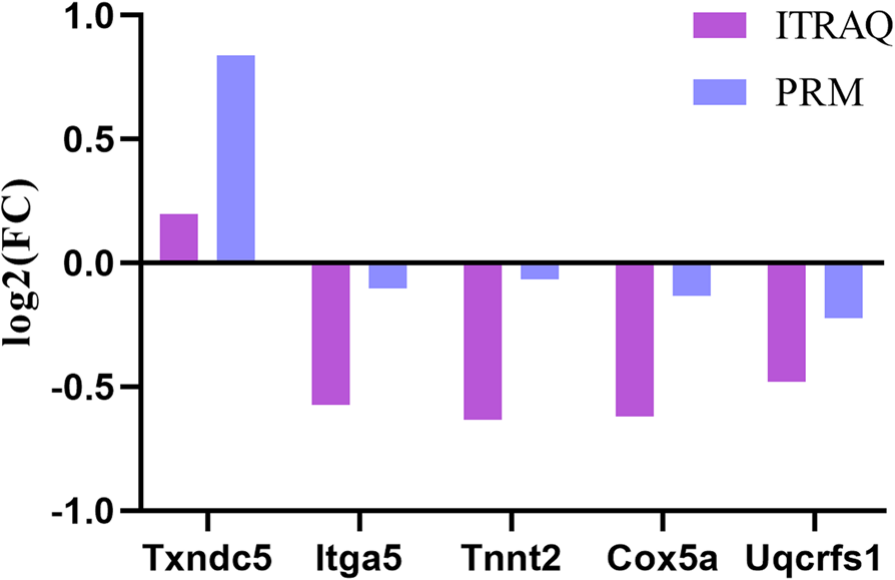
Comparison of protein expression by iTRAQ and PRM

A targeted proteomics technology with high resolution, high selectivity, and high sensitivity, PRM can detect the key protein specifically and has been widely used for protein quantification [19]. In this study, five DEPs, including RCG43947 (Txndc5, UniProt identifier D3ZZC1), Integrin alpha 5 (Itga5, UniProt identifier A0A0G2K1E1), Troponin T (Tnnt2, UniProt identifier F1LQ95), Cytochrome c oxidase subunit 5A (Cox5a, P11240), Cytochrome b-c1 complex subunit Rieske (Uqcrfs1, UniProt identifier P20788), they were randomly selected, and their expression was examined by PRM instead of the traditional “western blot”. The results showed that the up-regulation or down-regulation trends of the five proteins detected by PRM are consistent with those detected by iTRAQ,, indicating that iTRAQ was reliable and reproducible (Fig. 7).

### Bioinformatics analysis of key proteins

**Fig. 8:**
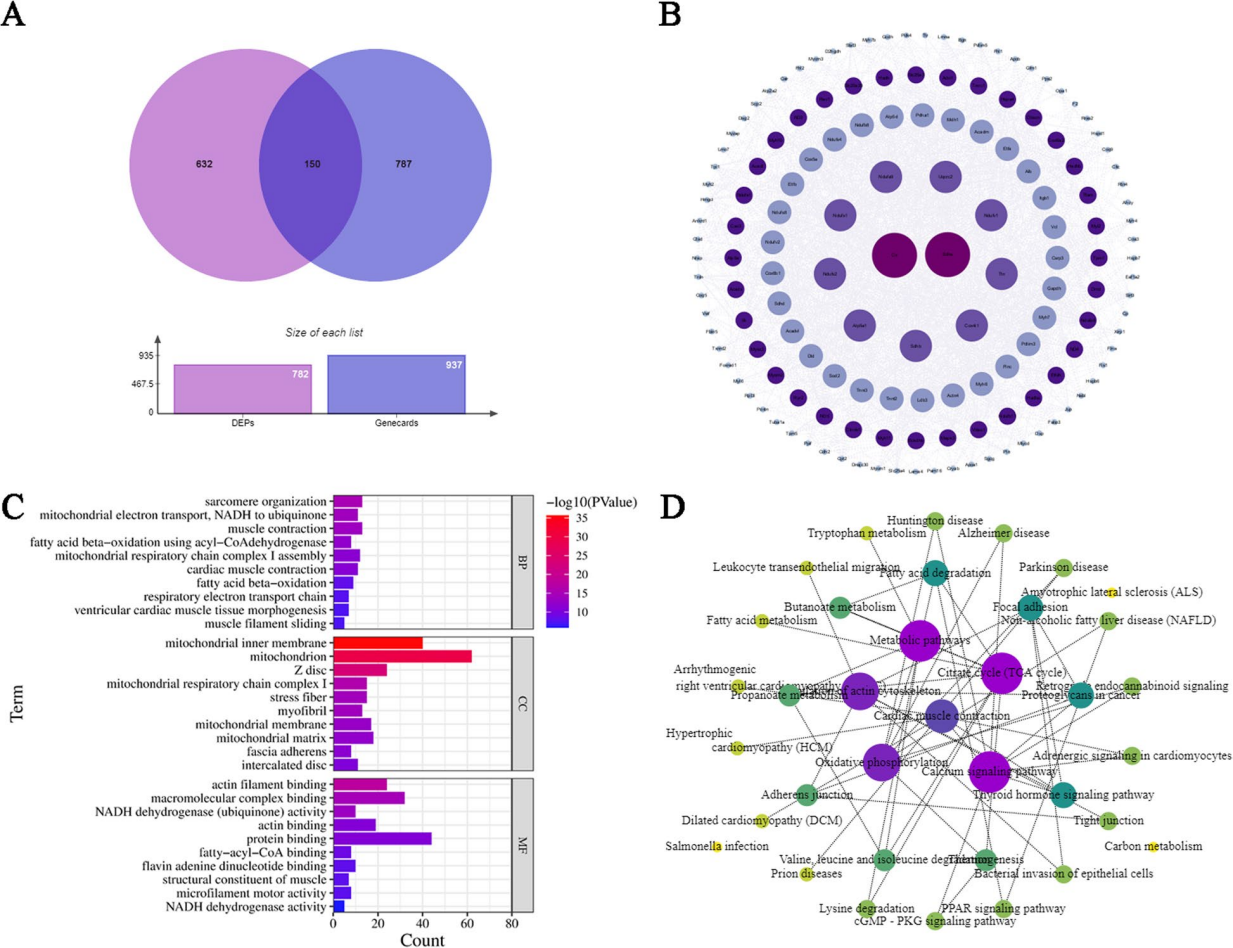
The functional annotation and enrichment analysis of the key proteins: (**A**) Venn diagram showing the overlapping key proteins. **B** PPI networks of key proteins. **C** The gene ontology (GO) analyses of key proteins. **D** KEGG enrichment pathways of key proteins

To further understand the interactions between key proteins, the STRING database was used to determine their protein-protein interactions (PPI) and Cyto-scape software was used to visualize the interactions. The size and color of the circles of the proteins in the network indicate node degree. The degree of nodes from the inner ring to the outer ring is this: 50–59, 40–49, 30–39, 20–29,10–19, 0–9 (Fig. 8B).

The GO analysis of key proteins was performed, sarcomere organization, mitochondrial electron transport, NADH to ubiquinone, muscle contraction, cardiac muscle contraction, ventricular cardiac muscle tissue morphogenesis, and muscle cell development were the primary functional categories in BP. A significant change has taken place in the mitochondria, the Z disc, mitochondrial respiratory chain complex I, and the myofibril in CC. Actin filament binding, actin binding, protein binding, and structural composition have changed in MF (Fig. 8C). Then, KEGG analyses were conducted and the results are shown in Fig. 8D. A majority of these key proteins were also associated with oxidative phosphorylation, cardiac muscle contraction, and Citrate cycle (TCA cycle). It was suggested by association analysis that the three pathways of oxidative phosphorylation, cardiac muscle contraction, and Citrate cycle (TCA cycle) and the proteins may play a key role in the pathogenesis of DCM.

## Materials and methods

### Data collection and preparation

GeneCards (http://www.genecards.org/) is a searchable, comprehensive database that integrates information on genes (GeneCards), diseases (MalaCards) and pathways (PathCards) from 150 sources. It is one of the largest human databases in the world. The keyword "dilated cardiomyopathy" was searched in the GeneCards database to obtain the target genes associated with DCM.

### Animals experiment 

#### The establishment of DCM model

In this study, 30 healthy male adult Sprague-Dawley rats (8 weeks old, weighing 250-300g at the beginning of the experiment) were utilized from the laboratory of Hainan Medical University (Haikou, China). The laboratory conditions were a 12/12 h light/dark cycle, 21–24 °C, and a pathogen-free environment. Water and food were available freely during all experiments. Rats were randomly allocated to one of two groups: Sham (*n* = 10) or DCM (*n* = 20). The DCM group, which received doxorubicin (DOX) by intravenous injection at a dose of 2.5 mg/kg once a week for eight weeks. The control group of rats received a weekly intravenous injection of saline (0.9% (9 g/L) NaCl) for up to eight weeks [20]. Echocardiography and N-terminal pro-brain natriuretic peptide (NT-proBNP) were used to detect and verify the development of DCM. At the end of the experiment, all rats were anesthetized by intraperitoneally injecting pentobarbital sodium (1%, 10 mL/100 g) before the sacrifice, and the hearts were preserved at -80°C before detection. Of the myocardial samples extracted, 9 were randomly selected for iTRAQ detection and 3 for PRM validation.

#### Echocardiographic evaluation

Ultrasonic cardiogram was monitored after discontinuation of DOX treatment everyone week, lasting for 4 weeks until the end of the study. An intraperitoneal injection of pentobarbital (100 mg/kg) anesthetized the animals and shaved their chests. Echocardiography was performed to evaluate cardiac morphology and function employing a Philips iE33xMATRIX echocardiography system (Philips Healthcare, Amsterdam, The Netherlands) equipped with an 8–12 MHz ultrasound probe. A parasternal short axis view at the level of papillary muscles was examined by M-mode echocardiography. The left ventricle major cardiac function-related parameters, containing the diameter of the left ventricle in diastole (LVIDd), and the diameter of the left ventricle in systole (LVIDs), left ventricular ejection fraction (EF) and fractional shortening (FS).

### ELISA

Rats' whole blood samples were collected into 1.5ml centrifuge tubes, and serum samples were obtained by centrifuging blood samples for 10 min at 3000 rpm, The serum samples were stored at -80°C until they were detected. N-terminal pro-brain natriuretic peptide (NT-proBNP) was determined by ELISA (ELISA kits were purchased from Huijia Biotech, Xiamen, China). The assays were conducted according to the manual instructions.

#### Hematoxylin‑eosin (HE) and Masson's staining

Rat hearts were taken and immediately immersed in 4% neutral PBS paraformaldehyde for 24 hours, 4% neutral PBS paraformaldehyde was immediately applied to rat myocardial tissues. Rat myocardial tissues were then immersed for 24 hours in the solution, then transferred to 70% ethanol, embedded in paraffin, sectioned into size of 5µm, and then dewaxed in xylene and dehydrated in ethanol. Finally, sections were stained with HE or Masson's trichrome for 5 min and observed under light microscopy for morphological changes in the myocardium and Fibrosis.

### Proteomic analysis

#### Sample preparation and iTRAQ labeling

Three randomly selected individuals from the same treatment group were randomly mixed to reduce individual differentiation. Nine samples from each group were subjected to protein extraction. Firstly, an appropriate amount of tissue samples was grinded in liquid nitrogen and the powder was transferred to 5 mL centrifuge tube, and then sonicated 15 minutes on ice bath using an ultrasonic processor in lysis buffer (7M Urea/2M Thiourea/4% SDS/40 mM Tris-HCl, pH 8.5/1mM PMSF/2mM EDTA) to extract total protein. After centrifugation at 4°C for 20 minutes at 12,000 rpm. Following addition of prechilled acetone and 30 mM DTT to the centrifuge tube, the mixture was precipitated at -20°C for 2 hours, centrifuged at 12000 rpm for 20 minutes at 4°C, then vacuum dried. As a final step, the protein concentration was determined using the Bradford method.

As directed by the manufacturer, the peptides mixture from each sample was labeled using the iTRAQ Reagent-8 plex Multiplex Kit (AB Sciex U.K. Limited). Samples were labelled as following: DCM-I (DCM1, DCM2 and DCM3), 115; DCM-II (DCM4, DCM5 and DCM6), 116; DCM-III (DCM7, DCM8 and DCM9), 117; Control-I (Ctrl-1, Ctrl-2 and Ctrl-3), 118; Control-II (Ctrl-4, Ctrl-5 and Ctrl-6), 119; Control- III (Ctrl-7, Ctrl-8 and Ctrl-9), 121. Then, the six labeled peptide samples were pooled and lyophilized using a vacuum concentrator.

#### LC and MS/MS analyses

The Polypeptide samples were analyzed with the Triple-TOF 5600 Plus liquid chromatography-mass spectrometer coupled with Eksigent nanoLC system (AB SCIEX, Massachusetts, USA). In brief, the labeled samples were reconstituted in reverse phase liquid chromatography (RPLC) mobile phases buffer (20mM ammonium formate in 2% acetonitrile, pH 10.0). Subsequently, the labeled samples were eluted at a flow rate of 1 ml/min for 50 min using a high-performance liquid chromatography (HPLC) system (Dinoex Ultal3000BioRS, Thermo Fisher, Waltham, MA, USA) equipped with Durashell C18 (5µm, 100 Å, 4.6×250 mm) for mobile phase elution of the samples. Next, the samples were eluted on a C18 trap column (5µm, 100µm×20mm) and a C18 analytical column (3µm, 75µm×150mm) with a flow rate gradient of 350 nl/min for 90 min. The two mobile phases were buffer A (0.1% (v/v) formic acid, 2% (v/v) acetonitrile, 98% H2O) and buffer B (0.1% (v/v) formic acid, 98% (v/v) acetonitrile, 2% H2O) Specifically. The final MS/MS scans were performed at a spray voltage of 2.3KV. MS1 spectra were collected for 250ms, followed by MS2 spectra for 50ms, and precursor ions were excluded from rescreening within 15s.

#### Proteomic data analysis and bioinformatics

The raw data for mass spectrometry analysis are RAW files, which were processed using the ProteinpilotTM V4.5 search engine (AB SCIEX, Massachusetts, USA) for protein identification and quantification. The retrieval parameters were set as follows: Triple TOF 5600 instrument system, trypsin, iTRAQ 8-plex quantification, biological modifications, cysteine modified with iodoacetamide, biological modifications were chosen as the subject of ID. More importantly, the database used for this analysis is the uniport-taxonomy _ Rattus _ norvegicus. Fasta (36153 sequences in total). For the identification results of Proteinpilot, only proteins with an unused value greater than 1.3 are taken into account. Meanwhile, a 95% confidence interval (CI) was set as the significance threshold for protein identification. Peptides were filtered utilizing 1% false discovery rate (FDR) and at least one unique. The ABI ProteinPilot software v4.5 were used to calculate iTRAQ ratios and *p*-values. In this study, proteins with a fold change ≥ 1.5 (or ≤ 0.67) and *p*-value < 0.05 as the thresholds were considered as differently expressed proteins.

Gene Ontology (GO) annotations for biological processes (BP), cellular components (CC), and molecular functions (MF) were performed using the DAVID online database (http://david.abcc.ncifcrf.gov). The plugin ClueGO in cyto-scape (version 3.7.1) is used to help researchers understand the biological pathways of differentially expressed proteins.

### Association analysis of key target genes and DEPs in DCM

The key genes obtained by data mining were imported into string database (https://string-db.org/) to obtain their corresponding proteins. The overlapping proteins of the two datasets were then identified as key proteins for DCM and visualized with the Venn diagram.

#### Parallel reaction monitoring (PRM) of proteins

PRM is a targeted proteomics technology with high-resolution, high selectivity and high sensitivity, which can detect the target protein purposefully [19]. The quaternary rod (Q1) of the target peptide is screened by mass spectrometer and transmitted to the collision chamber (Q2) for fragmentation, and all the fragment ions are scanned by the mass analyzer TOF and enter the detector to generate a high-resolution secondary mass spectrum (MS2spectrum). Finally, MS2spectrum can be searched in the database to achieve identification or confirmation, the mass spectrometry scanning mode of PRM is shown in the Figure S1.

Consequently, to verify the expression of DEPs, PRM was employed to further quantify the expression levels of the 5 DEPs in the remaining cryopreserved myocardial samples (*N* = 6, 3 in each group). Protein extraction, trypsin digestion and LC-MS/MS analysis are the same as iTRAQ experiments, except for PRM data acquisition. All the peptide samples after enzymatic hydrolysis were detected by mass spectrometry and then processed by ProteinPilotTMV4.5 search engine. The target peptide m/z was then added to the inclusion list to establish the method of mass spectrometry acquisition. Skyline software (https://skyline.ms/) was used to obtain quantitative information about the target proteins and peptides.

#### Bioinformatics analysis of key proteins

The key proteins were analyzed to obtain DCM-related biological function with the GO and KEGG. Finally, the differential proteins were imported into the STRING online database (http://string-db.org) for protein interaction analysis and visualized by Cyto-scape software.

### Statistical analysis

ANOVA was used to analyze differences between groups. All results in this study are presented as the mean ± SEM and were analyzed using SPSS 22.0 statistical software. The Tukey test was applied at the 5% probability level to distinguish statistically significant means.

## Discussion

DCM is a common cardiomyopathy worldwide, which is characterized by ventricular dilatation and systolic dysfunction [5]. A frequent cause of heart failure and sudden death, it causes sudden death due to heart failure [4]. As a result, our understanding of the molecular mechanism of DCM is still very limited and the pathogenesis and etiology remain unclear. Proteomics is currently used to study the cellular changes caused by diseases as well as the physiological changes induced by them [21]. It is reported that most of the research on the proteomics of heart disease focuses on human heart failure caused by DCM or ICM [22–24]. Whereas, DCM patients may be caused by different causes, including genetic mutations, genetic mutations, environmental influences, viral infections, and cardiologic substances and other unknown factors [1]. Therefore, Proteomic studies of the human hearts are difficult to reveal the precise pathophysiological mechanism of a specific etiology. Here, an animal model with better homogeneity is used to conduct this study in order to provide more accurate information for further human research. As part of this study, iTRAQ coupled MS/MS was utilized to detect rat myocardial tissue and examine protein expression changes to reveal physiological/pathological mechanisms of DCM. In order to reduce biological variation and statistical differences, three myocardial samples from each group were mixed together, and then repeated three times [25]. Proteins with expression ratios of over 1.5-fold increase or at least 0.67-fold decrease while adj *p*-value < 0.05 were considered to be differentially expressed. As a consequence of the analysis, 782 DEPs were identified, including 348 proteins that were up-regulated and 434 proteins that were down-regulated. To understand the detailed mechanism of pathogenesis of DCM, we further performed an association analysis of DEPs and target genes. In the present study, the function of DEPs was further analyzed and discussed using GO enrichment and KEGG pathway analysis indicated that proteins involved in processes such as Citrate cycle (TCA cycle), Oxidative phosphorylation, Cardiac muscle contraction may play a key role in DCM.

### DEPs involved in TCA cycle

TCA is the common metabolic pathway and connection hub of the three nutrients, and it is an important step for mitochondria to generate energy. Seven mitochondrial proteins are associated with the TCA cycle and are involved in ATP synthesis, according to our data. It is reported that more than 90% of the ATP used by cardiomyocytes for contraction is produced by mitochondrial respiration [26]. PDHA and DLD are the components of pyruvate dehydrogenase complex (PDH), PDH catalyzes the oxidative decarboxylation of pyruvate to acetyl-CoA in mitochondria to regulate glycolysis products into the tricarboxylic acid cycle. Furthermore, PDHA and DLD are components of the pyruvate dehydrogenase complex (PDHC). The role of PDHC is to catalyze the oxidative decarboxylation of pyruvate in mitochondria to acetyl-CoA, which plays an important role in the energy metabolism of mitochondrial respiratory chain [27]. TCA's rate-limiting enzyme, Cs, catalyzes the conversion of acetyl-CoA to citric acid [28]. In our study, it was found that the protein markers involved in TCA cycle were significantly down-regulated, indicating that mitochondrial dysfunction was involved in the occurrence and development of DCM, and energy metabolism played an important role in DCM. In spite of previous studies have shown that the changes in the proteins and metabolites involved in energy metabolism [29, 30]. In our research, we have examined the specific changes of these markers, providing further proves that the TCA cycle disorder caused the occurrence of DCM. The abnormal mitochondria in myocardial cells result in insufficient energy supply to the tissue, resulting in cardiomyocyte remodeling and eventually cardiomyocyte dysfunction.

### DEPs involved in oxidative phosphorylation

The primary physiological function of mitochondria is to generate ATP through oxidative phosphorylation [31]. The effective supply of myocardial energy is dependent on mitochondrial oxidative phosphorylation, and mitochondrial oxidative phosphorylation depends on the respiratory chain function of mitochondrial inner membrane [32]. The mitochondrial respiratory chain consists of four polypeptide complexes (I-IV), ubiquinone and CytC, while NADH, SDH, CYC1, COX5A and PPA2 are important components of complex I, II, III, IV and ATP synthase, respectively. It has been reported that heart failure has been associated with impaired mitochondrial oxidative phosphorylation caused by the disturbance of electron transport, the hindrance of oxidation, the lack of energy needed for phosphorylation or the disconnection between electron transport and phosphorylation, although there is energy produced by oxidation, it cannot be converted into ATP [33]. An important finding of this study was the significant reduction in the amount of related proteins involved in the oxidative phosphorylation process, indicating that the oxidative modified proteins involved in the electron transport chain may cause abnormal metabolism of cardiomyocytes, which may directly affect the energy supply of cardiomyocytes. Moreover, the abnormal metabolism of cardiomyocytes may be related to reactive oxygen species generated by the respiratory chain's unbalanced activities that damage cardiomyocytes. A number of previous studies have demonstrated a decrease in respiratory chain complex I activity as well as ATP synthase activity, but SDHA activity did not change in mouse models of human DCM and other cardiomyopathies [34]. In addition, DCM also decreased the enzyme activities of cytochrome complexes III and IV of the mitochondrial respiratory chain, while the activities of complexes II and ATP synthase had no significant change [35]. Alternatively, our study found that the enzyme activities of the entire respiratory chain complex of DCM mitochondria were significantly reduced, rather than limited to some of them. This not only confirmed the previous research, but also further demonstrates that the mitochondrial respiratory chain complex consists of NADH, SDH, CYC1, COX5A, and PPA2, which play an important role in oxidative phosphorylation. In other words, damage to any component of the mitochondrial respiratory chain may affect myocardial energy production and ultimately result in DCM.

### DEPs involved in cardiac muscle contraction

Following depolarization of the surface membrane of the heart, excitatory-contraction coupling results in the release of calcium ions from the sarcoplasmic reticulum and myocardial contraction. Research on the expression and activity changes of various proteins involved in the release and reabsorption of calcium from myocardial sarcoplasmic reticulum, reabsorption and storage constitute the main content of human heart failure research [36]. It was found that 16 proteins were involved in cardiac muscle contraction of DCM, of which 7 proteins displayed differential expression in the expression profile. In the expression profile, we can see that RyR2 and TnnT2 proteins were significantly down-regulated. According to previous studies, ryanodine receptors (RyRs) mRNA expression decreased significantly in DCM, and the protein expression did not change significantly in DCM, but the binding characteristics of ryanodine were changed [37]. Interestingly, some researchers have observed a decrease in the number and activity of RyRs in a canine heart failure model [38]. Our study revealed that RyR2 is significantly down-regulated in DCM, consequently, whether the expression and binding conformation of RyR2 have changed needs to be verified by further experiments.

In previous studies, mutations in genes such as TPM1, MYH7 and TnnT2 have been shown to be common causes of DCM and HCM [39, 40]. The mutation of TnnT2 gene in DCM reduced the sensitivity of troponin complex to Ca^2+^, reducing the contractility of the heart [40]. In addition, TPM1 is essential for the regulation and stability of actin during muscle contraction [41]. In our study, it was found that TnnT2 was significantly down-regulated, while TPM1 was significantly up-regulated, suggesting that reduced expression of RyR2 indirectly results in decreased Ca^2+^ release in the sarcoplasmic reticulum. Consequently, calcium homeostasis changes in cardiomyocytes may trigger remodeling of DCM cardiomyocytes and subsequently result in myocardial contractile dysfunction.

## Conclusion

In summary, we established a rat model of DCM. Thereafter we elucidated the key proteins and pathways of DCM by integrating data mining, iTRAQ-PRM proteomics and bioinformatics analysis. Firstly, DCM disease target genes were downloaded from public databases, and 935 genes were identified as key target genes. Next, a total of 782 DEPs, including 348 up-regulated and 434 down-regulated proteins, were identified in our animal experiment. The functional annotation of these DEPs revealed complicated molecular mechanisms including oxidation-reduction process, tricarboxylic acid cycle, protein folding, and triggered a series of molecular pathways involving TCA cycle, Oxidative phosphorylation, Cardiac muscle contraction. Finally, the DEPs were analyzed for association with the target genes screened in the public dataset. The overlapping proteins were validated by parallel reaction monitoring (PRM). We obtained 150 key proteins and further determined the importance of these three pathways. The results may provide a new insight for the detail mechanism of DCM and help to identify potential biomarkers associated with DCM.

### Supplementary Information

**Additional file 1: Table S1. **The differentially expressed proteins in the myocardial tissue between the DCM and control group, based on iTRAQ data. **Table S2. **A total of 154 overlapping proteins in DCM.

**Additional file 2: Figure S1. **PRM workflow: the skyline platform supports PRM-based targeted MS Quantification.

## Data Availability

The datasets supporting this article have been have been deposited to the ProteomeXchange Consortium via the PRIDE partner repository with the dataset identifier PXD038946. The direct link to the database is http://www.ebi.ac.uk/pride, Username: reviewer_pxd038946@ebi.ac.uk, Password: 19VIXeiP.
