# Supplementary material for: Insight into the underlying molecular mechanism of dilated cardiomyopathy through integrative analysis of data mining, iTRAQ-PRM proteomics and bioinformatics
Source: Proteome Sci. 2023 Sep 22;21:13. doi: 10.1186/s12953-023-00214-9 (PMC10517512; doi:10.1186/s12953-023-00214-9)
Supplement: Supplementary file 1 — Additional file 1: Table S1. The differentially expressed proteins in the myocardial tissue between the DCM and control group, based on iTRAQ data. Table S2. A total of 154 overlapping proteins in DCM. [file 12953_2023_214_MOESM1_ESM.docx]

| Protein ID | GeneSymbol | Fold change (DCM/Control) | Regulation |
| --- | --- | --- | --- |

**Table S1 The differentially expressed proteins in the myocardial tissue between the DCM and control group, based on iTRAQ data**

| A1L114 | Fga | 8.34036154217191 | UP |
| --- | --- | --- | --- |
| A0A0G2K8Q1 | Apoc3 | 8.20535776350233 | UP |
| Q63041 | A1m | 8.01338137520684 | UP |
| P14480 | Fgb | 7.42903791533577 | UP |
| M0R8B6 | Tubb1 | 7.2577412393358 | UP |
| P14630 | Apom | 6.74441819720798 | UP |
| Q5PQU1 | Kng2 | 6.72938913769192 | UP |
| P55797 | Apoc4 | 6.70677134725783 | UP |
| F1LNY3 | Ncam1 | 6.65764782163832 | UP |
| B0BNA5 | Cotl1 | 6.60242884688907 | UP |
| P19939 | Apoc1 | 6.08690611521403 | UP |
| D3Z802 | Nrap | 5.96535136964586 | UP |
| Q5RK09 | Eif3g | 5.60358635584513 | UP |
| P04762 | Cat | 5.57840710216098 | UP |
| G3V8R1 | Nucb2 | 5.47136301464505 | UP |
| Q66HH8 | Anxa5 | 5.41499013370938 | UP |
| D3ZH41 | Ckap4 | 5.25912862353855 | UP |
| A0A140TAI1 | Ubqln1 | 5.05658769607544 | UP |
| P05942 | S100a4 | 4.85976084073385 | UP |
| M0RCF7 | LOC100909761 | 4.82438770929972 | UP |
| Q6P792 | Fhl1 | 4.7820783191257 | UP |
| P86182 | Ccdc22 | 4.65448252360026 | UP |
| G3V8D4 | Apoc2 | 4.64153830210368 | UP |
| A0A140TAE0 | Mvp | 4.58258864614699 | UP |
| P02680 | Fgg | 4.48154844178094 | UP |
| F1LPM3 | Sorbs2 | 4.19147189458212 | UP |
| A0A0G2JSM3 | Pdlim3 | 4.12320196628571 | UP |
| P04785 | P4hb | 4.10195305612352 | UP |
| D4ABA9 | Xirp1 | 4.05154222912259 | UP |
| A0A0H2UHR7 | Flnc | 4.02099582884047 | UP |
| F1M865 | Sorbs1 | 3.99392088254293 | UP |
| Q8R3Z7 | Ehd4 | 3.97667739126417 | UP |
| B5DF63 | Slmap | 3.96987904442681 | UP |
| G3V852 | Tln1 | 3.89645897017585 | UP |
| P52631 | Stat3 | 3.8580293920305 | UP |
| B6DYP8 | Gsta1 | 3.80754199292925 | UP |
| Q6P742 | Plp2 | 3.7613090078036 | UP |
| F1MAN8 | Lama5 | 3.68272373411391 | UP |
| Q6P7S6 | Clu | 3.58878967497084 | UP |
| G3V778 | Pdk4 | 3.5478585296207 | UP |
| Q9ER24 | Atxn10 | 3.4676446252399 | UP |
| P07323 | Eno2 | 3.37542127238379 | UP |
| P69897 | Tubb5 | 3.33661227756076 | UP |
| A0A0G2JSZ5 | Pdia6 | 3.32093509038289 | UP |
| F1M6Z1 | Apob | 3.28613670667013 | UP |
| P82995 | Hsp90aa1 | 3.27341741985745 | UP |
| Q6P6R6 | Tgm2 | 3.27212121751573 | UP |
| Q5PPG2 | Lgmn | 3.2512801223331 | UP |
| F1LYX9 | Dsg2 | 3.20354607370165 | UP |
| A0A0G2K568 | Crym | 3.19969720310635 | UP |
| B1WC33 | Cdc42ep4 | 3.15389204025269 | UP |
| B6DYQ7 | Gstp1 | 3.14555079407162 | UP |
| F1LR02 | Col18a1 | 3.1402772532569 | UP |
| G3V7U0 | Csrp3 | 3.12674122386509 | UP |
| Q03626 | Mug1 | 3.12406680319044 | UP |
| Q810D0 | DMN | 3.06797819667392 | UP |
| G3V913 | Hspb1 | 3.06326030360328 | UP |
| P23928 | Cryab | 3.06163342793783 | UP |
| C5NMH2 | LOC100909784 | 3.05113901032342 | UP |
| Q7M0E3 | Dstn | 3.04963901307848 | UP |
| Q6P0K8 | Jup | 3.04002888997396 | UP |
| B2GVB1 | S100a6 | 3.02966062227885 | UP |
| P31000 | Vim | 2.98202964994643 | UP |
| Q5XI38 | Lcp1 | 2.97924248377482 | UP |
| P04905 | Gstm1 | 2.97495928075579 | UP |
| Q6GMN8 | Actn1 | 2.97337714831034 | UP |
| P27605 | Hprt1 | 2.96086804072062 | UP |
| F1LQ48 | Hnrnpl | 2.94487170378367 | UP |
| P68370 | Tuba1a | 2.93094746271769 | UP |
| F1LQN3 | Rtn4 | 2.8961593442493 | UP |
| Q5I2Z0 | Pacsin3 | 2.8499538898468 | UP |
| G3V6S3 | Calu | 2.83429521984524 | UP |
| E9PTU4 | Myh11 | 2.8175534274843 | UP |
| A0A0H2UHM5 | Pdia3 | 2.80657741758558 | UP |
| P55159 | Pon1 | 2.80210732751422 | UP |
| Q66HL0 | Nt5e | 2.77067976527744 | UP |
| B5DFG4 | Hspb7 | 2.74747241867913 | UP |
| F1M957 | Vwf | 2.74631817473306 | UP |
| P07150 | Anxa1 | 2.72806644439697 | UP |
| F1LNF1 | Hnrnpa2b1 | 2.72734765211741 | UP |
| R9PXU6 | Vcl | 2.72133677535587 | UP |
| Q07936 | Anxa2 | 2.71383942498101 | UP |
| O35802 | ITIH4 | 2.70564454131656 | UP |
| B0BNA7 | Eif3i | 2.704736272494 | UP |
| P59215 | Gnao1 | 2.70455574989319 | UP |
| A0A0G2JZT5 | Sept7 | 2.67399020989736 | UP |
| Q6TUG0 | Dnajb11 | 2.67002635531955 | UP |
| Q91Y81 | Septin2 | 2.66904675298267 | UP |
| Q920J4 | Txnl1 | 2.65736989180247 | UP |
| G3V6T7 | Pdia4 | 2.65698843532138 | UP |
| Q6URK4 | Hnrnpa3 | 2.62575099203322 | UP |
| D3ZBS2 | Itih3 | 2.62106266286638 | UP |
| P62630 | Eef1a1 | 2.62044110563066 | UP |
| B2GVB9 | Fermt3 | 2.60848479800754 | UP |
| P13084 | Npm1 | 2.59886601236131 | UP |
| B0BN97 | Txndc12 | 2.59875399536557 | UP |
| A0A0G2K1E2 | Itga5 | 2.5617034567727 | UP |
| Q5M891 | C4bpa | 2.54601707061132 | UP |
| Q569B3 | Igh-6 | 2.52168252733019 | UP |
| O70593 | Sgta | 2.51282926400503 | UP |
| Q9ER30 | Klhl41 | 2.51120865345001 | UP |
| B2GV73 | Arpc3 | 2.48464365800222 | UP |
| Q63413 | Ddx39b | 2.48111444049411 | UP |
| A0A0G2K586 | Fabp4 | 2.47500859366523 | UP |
| D3ZUL3 | Col6a1 | 2.47330779499478 | UP |
| B4F795 | Slc44a2 | 2.43825876712799 | UP |
| G3V912 | Tmx4 | 2.42906590302785 | UP |
| D3ZRM0 | Dusp27 | 2.42771754662196 | UP |
| F1LMV6 | Dsp | 2.42628524038527 | UP |
| Q924L6 | Mdr1a | 2.41629593902164 | UP |
| F1M779 | Cltc | 2.39287430710263 | UP |
| D3Z9E1 | Emilin1 | 2.38315676318275 | UP |
| P47853 | Bgn | 2.37684945265452 | UP |
| P06761 | Hspa5 | 2.36758522192637 | UP |
| Q5U328 | Ncl | 2.36456533273061 | UP |
| G3V624 | Coro1c | 2.358950442738 | UP |
| A0A0H2UHK5 | Lnpep | 2.32875835895538 | UP |
| B2RYG2 | Pck2 | 2.31429857677884 | UP |
| P07687 | Ephx1 | 2.31239354610443 | UP |
| Q4QQV0 | Tubb6 | 2.30947669347127 | UP |
| Q5M7U6 | Actr2 | 2.28603055742052 | UP |
| G3V7U2 | Map1a | 2.27502575185564 | UP |
| Q5BJU0 | Rras2 | 2.25802822907766 | UP |
| D3ZFH5 | Itih2 | 2.25517282221052 | UP |
| Q8R560 | Ankrd1 | 2.24781941043006 | UP |
| F1LNH3 | Col6a2 | 2.23406487041049 | UP |
| Q6P6G9 | Hnrnpa1 | 2.2337964243359 | UP |
| A0A0H2UH90 | Vwa5a | 2.23249442047543 | UP |
| F1MAA7 | Lamc1 | 2.23089513513777 | UP |
| P52944 | Pdlim1 | 2.22912398974101 | UP |
| Q07205 | Eif5 | 2.21756635109584 | UP |
| Q8R4A1 | Ero1a | 2.21138448185391 | UP |
| D3ZRE3 | Csnk1a1 | 2.17827880382538 | UP |
| Q9JKB7 | Gda | 2.17036966482798 | UP |
| F1LQ55 | Scp2 | 2.16177230411106 | UP |
| O35878 | Hspb2 | 2.15943633185493 | UP |
| G3V6P7 | Myh9 | 2.15703822506799 | UP |
| P60892 | Prps1 | 2.15021080440945 | UP |
| A0A0G2K013 | Actn4 | 2.14843099647098 | UP |
| Q6P7A7 | Rpn1 | 2.14034675227271 | UP |
| A0A0G2K435 | Dnajc7 | 2.13977199130588 | UP |
| Q641Z6 | Ehd1 | 2.13310596677992 | UP |
| F1LS79 | Cspg4 | 2.12282739082972 | UP |
| Q6P685 | Eif2s2 | 2.11545589235094 | UP |
| Q63610 | Tpm3 | 2.11106989118788 | UP |
| M0R6K0 | Lamb2 | 2.10904178354475 | UP |
| Q4G061 | Eif3b | 2.10279142856598 | UP |
| Q9Z270 | Vapa | 2.09499657154083 | UP |
| G3V6T1 | Copa | 2.0862035089069 | UP |
| Q5XIP0 | Dnajb4 | 2.07684290409088 | UP |
| Q6AYC4 | Capg | 2.07631822427114 | UP |
| P10111 | Ppia | 2.0719582107332 | UP |
| G3V9Y1 | Myh10 | 2.0521185596784 | UP |
| Q5M860 | Arhgdib | 2.04369213845995 | UP |
| G3V8L3 | Lmna | 2.04164588451385 | UP |
| A0A0G2JT93 | Ctnnb1 | 2.03893955548604 | UP |
| A0A0G2JSK5 | Itgb1 | 2.02367566691505 | UP |
| M0R907 | Snrpd3 | 2.02282279729843 | UP |
| Q3B7D0 | Cpox | 2.01483575503031 | UP |
| Q9Z1Y3 | Cdh2 | 2.01377432876163 | UP |
| P53987 | Slc16a1 | 2.01363242997063 | UP |
| A0A0G2JUA5 | Ahnak | 2.01209599441952 | UP |
| A0A387KC71 | Akr1c15 | 2.01073075665368 | UP |
| P04639 | Apoa1 | 18.6980391608344 | UP |
| P06238 | A2m | 18.1317620807224 | UP |
| Q5UAJ6 | COX2 | 12.9243129624261 | UP |
| P04638 | Apoa2 | 12.3521228896247 | UP |
| P23606 | Tgm1 | 11.740365518464 | UP |
| A0A097BW25 | Postn | 11.0645656585693 | UP |
| F1LMC2 | Xirp2 | 10.3719197379218 | UP |
| B5DFB2 | Rbbp4 | 1.98878790272607 | UP |
| P14841 | Cst3 | 1.98560976982117 | UP |
| A0A0G2K890 | Ezr | 1.98478321234385 | UP |
| G3V834 | Prrc1 | 1.97971816857656 | UP |
| D4A6C5 | Arhgap1 | 1.97736121548547 | UP |
| Q63544 | Sncg | 1.9738439851337 | UP |
| Q6PCT9 | Psmd6 | 1.96618591414558 | UP |
| A0A0G2K9T4 | Macf1 | 1.9655290974511 | UP |
| G3V6S0 | Sptbn1 | 1.96107325288984 | UP |
| C0JPT7 | Flna | 1.96078198485904 | UP |
| O08618 | Prpsap2 | 1.95738608307308 | UP |
| P46462 | Vcp | 1.95162231392331 | UP |
| D3ZZR9 | Fkbp2 | 1.95101277695762 | UP |
| Q6AYD3 | Pa2g4 | 1.9508767525355 | UP |
| F1M1B3 | Washc5 | 1.94731378555298 | UP |
| F1M155 | Svil | 1.94536088572608 | UP |
| Q9Z1H9 | Cavin3 | 1.94473499721951 | UP |
| F1MAQ7 | Son | 1.94170369042291 | UP |
| Q6MG61 | Clic1 | 1.93728544976976 | UP |
| B5DFC8 | Eif3c | 1.92326949040095 | UP |
| A0A096MJB5 | Unc45b | 1.92233751879798 | UP |
| D4A8F2 | Rsu1 | 1.92227925194634 | UP |
| Q794E4 | Hnrnpf | 1.92000199688805 | UP |
| M0R735 | Syncrip | 1.91986420419481 | UP |
| P35565 | Canx | 1.91973989539676 | UP |
| P97541 | Hspb6 | 1.91857843928867 | UP |
| D3ZL10 | Col6a6 | 1.91695221265157 | UP |
| B2RZ37 | Reep5 | 1.91409554746416 | UP |
| D3ZVR9 | Pgm5 | 1.90818201171027 | UP |
| Q9EQP5 | Prelp | 1.90728109412723 | UP |
| B0BN18 | Pfdn2 | 1.89858775668674 | UP |
| A0A0A0MXY5 | Pfkp | 1.89340925216675 | UP |
| P47942 | Dpysl2 | 1.89181945721308 | UP |
| P01041 | Cstb | 1.89114389154646 | UP |
| A0A140TAA4 | Pdcd6ip | 1.88926532533434 | UP |
| I6L9G6 | Tardbp | 1.88486422432794 | UP |
| Q3MIE4 | Vat1 | 1.88390322526296 | UP |
| Q5RJR9 | Serpinh1 | 1.88271493381924 | UP |
| A0A0G2JUP3 | Obscn | 1.88143086433411 | UP |
| Q9WVH8 | Fbln5 | 1.88118678993649 | UP |
| P61983 | Ywhag | 1.87739643785689 | UP |
| Q6AYV6 | Fth1 | 1.87730880578359 | UP |
| Q6Q0N1 | Cndp2 | 1.87488823466831 | UP |
| D4A2G9 | Ranbp1 | 1.87389323446486 | UP |
| M0R9X8 | Dync1h1 | 1.87170433998108 | UP |
| Q6DGG0 | Ppid | 1.86297461059358 | UP |
| O08557 | Ddah1 | 1.86160622702704 | UP |
| F1LPS8 | Pura | 1.86156186792586 | UP |
| Q99J82 | Ilk | 1.86136489444309 | UP |
| D4AB17 | Pfas | 1.8548692199919 | UP |
| Q9EQS0 | Taldo1 | 1.84796277681987 | UP |
| Q6AYT3 | Rtcb | 1.84639088312785 | UP |
| G3V7C6 | Tubb4b | 1.84385367234548 | UP |
| A0A0G2KA25 | Tm9sf4 | 1.84235289361742 | UP |
| Q64240 | Ambp | 1.83641061517927 | UP |
| P21708 | Mapk3 | 1.83357090420193 | UP |
| F1LPQ9 | Akap2 | 1.8311789698071 | UP |
| F1M978 | Impa1 | 1.83061967955695 | UP |
| Q1W176 | Rmnd1 | 1.82970456944571 | UP |
| Q6AYF2 | Lmcd1 | 1.82748666074541 | UP |
| M0RDJ4 | Gmfb | 1.81325765450796 | UP |
| P25113 | Pgam1 | 1.80971810552809 | UP |
| Q5XI73 | Arhgdia | 1.80389246675703 | UP |
| D4A9L2 | Srsf1 | 1.79963545004527 | UP |
| D3ZUY8 | Ap2a1 | 1.79772423373328 | UP |
| A0A0A0MY09 | Hsp90b1 | 1.79727984799279 | UP |
| F1LMQ3 | Psmd8 | 1.79494610759947 | UP |
| G3V7G6 | Hspb3 | 1.79418655236562 | UP |
| D4A1R8 | Cpne1 | 1.78730762667126 | UP |
| E9PT82 | Strn3 | 1.77321357197232 | UP |
| Q6NYB7 | Rab1A | 1.77066445350647 | UP |
| P51635 | Akr1a1 | 1.77020200093587 | UP |
| F1LM60 | Arap1 | 1.76232428020901 | UP |
| Q02759 | Alox15 | 1.75399061706331 | UP |
| Q6AYK3 | Isyna1 | 1.75015764766269 | UP |
| Q5U300 | Uba1 | 1.74932743443383 | UP |
| Q5XIF6 | Tuba4a | 1.74566211965349 | UP |
| P24368 | Ppib | 1.73915731906891 | UP |
| B2RYV0 | Dnajc30 | 1.73703791035546 | UP |
| B2RZD1 | Sec61b | 1.73504364490509 | UP |
| Q5M878 | Saa4 | 1.73420029878616 | UP |
| Q6AY58 | Bcap31 | 1.73275734318627 | UP |
| Q5RKI5 | Flii | 1.7284720076455 | UP |
| D3ZJZ0 | Tmem205 | 1.72693933380975 | UP |
| Q920Q0 | Palm | 1.72553367084927 | UP |
| F1LQS6 | Xdh | 1.71924257278442 | UP |
| Q4KMA2 | Rad23b | 1.70498724778493 | UP |
| A0A0G2JTA0 | Faf2 | 1.7046404282252 | UP |
| A0A0G2JV31 | Xpnpep1 | 1.70433411333296 | UP |
| A1L108 | Arpc5l | 1.7005962729454 | UP |
| P18418 | Calr | 1.70014446311527 | UP |
| P32198 | Cpt1a | 1.69611542092429 | UP |
| Q4FZT9 | Psmd2 | 1.69510099622938 | UP |
| Q497C9 | Mfap4 | 1.68930378225115 | UP |
| D3ZZU4 | Tmem160 | 1.68721009625329 | UP |
| F1M8Z8 | Sorbs1 | 1.68673735194736 | UP |
| Q5XI86 | Ptrh2 | 1.68463542064031 | UP |
| G3V7L0 | Fdx1 | 1.68393667538961 | UP |
| P81795 | Eif2s3 | 1.68343107567893 | UP |
| Q6IMZ3 | Anxa6 | 1.67436863978704 | UP |
| B1WBM0 | Cd9 | 1.67145033677419 | UP |
| P62963 | Pfn1 | 1.67093022664388 | UP |
| Q5VJM4 | Ttn | 1.67061428891288 | UP |
| P05708 | Hk1 | 1.66450709766812 | UP |
| D4AC23 | Cct7 | 1.66418778896332 | UP |
| Q1PBJ1 | Mfge8 | 1.66389567322201 | UP |
| D3ZJB1 | Mfap5 | 1.66155245569017 | UP |
| Q9Z0U8 | pRM10 | 1.65916465388404 | UP |
| Q9JJ54 | Hnrnpd | 1.65906180275811 | UP |
| F8WFH8 | Wars | 1.65797754791048 | UP |
| D4A4D5 | LOC498555 | 1.65279319551256 | UP |
| Q5HZE2 | Tmem120a | 1.64735129806731 | UP |
| G3V7Z3 | Nol3 | 1.6398878759808 | UP |
| Q6P6U2 | Psmc3 | 1.63498075803121 | UP |
| P85973 | Pnp | 1.63297465112474 | UP |
| A4GW50 | Stk38l | 1.63150687350167 | UP |
| Q64119 | Myl6 | 1.62750089168548 | UP |
| G3V8A5 | Vps35 | 1.627166112264 | UP |
| A0A0G2K2S2 | Slc2a1 | 1.62681894169913 | UP |
| Q6P6T6 | Ctsd | 1.62605779700809 | UP |
| E9PT79 | Tsn | 1.6249909930759 | UP |
| D4A8G5 | Tgfbi | 1.62485411432054 | UP |
| D4A9D6 | Dhx9 | 1.62139154805078 | UP |
| D4A8D5 | Flnb | 1.62029163042704 | UP |
| P84079 | Arf1 | 1.62009154425727 | UP |
| Q6IUR5 | Nenf | 1.61816390355428 | UP |
| P05197 | Eef2 | 1.61757798989614 | UP |
| P06765 | Pf4 | 1.61467111110687 | UP |
| Q641X8 | Eif3e | 1.6139061583413 | UP |
| Q6JBI7 | Lmo7 | 1.61281866497464 | UP |
| F1LN42 | Tns1 | 1.61208642853631 | UP |
| Q5XFX0 | Tagln2 | 1.61174088054233 | UP |
| Q5EB94 | Myzap | 1.60599055555132 | UP |
| G3V8B6 | Psmd1 | 1.60360957516564 | UP |
| Q566D5 | Eif1a | 1.60211377673679 | UP |
| D3ZQN7 | Lamb1 | 1.60102866755592 | UP |
| P10760 | Ahcy | 1.5966186788347 | UP |
| D3ZZV1 | Pam16 | 1.59651279449463 | UP |
| Q63002 | M6P/IGF2r | 1.59306821558211 | UP |
| D4A720 | Srsf7 | 1.58956066767375 | UP |
| G3V829 | Fubp3 | 1.58854483895832 | UP |
| Q5XID6 | Sgcg | 1.58612877792782 | UP |
| A0A0G2JU77 | Eif3k | 1.58232135242886 | UP |
| D3ZZC1 | Txndc5 | 1.58116017447578 | UP |
| D3ZMY7 | Nt5c2 | 1.58029021819433 | UP |
| D4AAE9 | Cisd2 | 1.57540600167381 | UP |
| Q4KLZ0 | Vnn1 | 1.57518765661452 | UP |
| P00786 | Ctsh | 1.57311343484455 | UP |
| Q5XI72 | Eif4h | 1.56866447793113 | UP |
| A0A1W2Q6E9 | Msn | 1.56821611192491 | UP |
| D3ZVB7 | Ogn | 1.56569670306312 | UP |
| Q3T1L0 | Aldh16a1 | 1.56513755851322 | UP |
| R9PXU4 | Txnrd1 | 1.56151321861479 | UP |
| Q63570 | Psmc4 | 1.55934777524736 | UP |
| Q5M7W5 | Map4 | 1.55815369553036 | UP |
| D4ACM1 | Elp3 | 1.55790579319 | UP |
| G3V6E1 | Myh2 | 1.55496921804216 | UP |
| Q4G022 | Get3 | 1.55429210927751 | UP |
| P50137 | Tkt | 1.5491551955541 | UP |
| Q5XIE0 | Anp32e | 1.54859167999692 | UP |
| P51638 | Cav3 | 1.54828655719757 | UP |
| F1LV13 | Hnrnpm | 1.54576557212406 | UP |
| A0A0H2UHQ3 | Map1s | 1.54159710142348 | UP |
| Q6RUV5 | Rac1 | 1.53872195217345 | UP |
| P45479 | Ppt1 | 1.53753834300571 | UP |
| P09895 | Rpl5 | 1.53457412454817 | UP |
| P62907 | Rpl10a | 1.53425143824683 | UP |
| Q66H80 | Arcn1 | 1.53225760989719 | UP |
| O08949 | Gtf2a1 | 1.53165868918101 | UP |
| G3V843 | F2 | 1.53140766090817 | UP |
| P52481 | Cap2 | 1.52991131941478 | UP |
| B0BN74 | Bag2 | 1.52865874767303 | UP |
| F1LRV4 | Hspa4 | 1.52659728129705 | UP |
| D3ZD73 | Ddx6 | 1.52503089110057 | UP |
| P97852 | Hsd17b4 | 1.52492405308617 | UP |
| P62859 | Rps28 | 1.51816060145696 | UP |
| P62909 | Rps3 | 1.51479722393884 | UP |
| O35509 | Rab11b | 1.51410820749071 | UP |
| Q99ML5 | Pcyox1 | 1.50821900367737 | UP |
| P63322 | Rala | 1.50767262114419 | UP |
| B1WBS4  A0A096MJ01 | Vps26b  Ldb3 | 1.50427834855186  1.50324022769928 | UP  UP |
| F1LTF8 | Lama4 | 1.50277225176493 | UP |
| Q91ZW1 | Tfam | 0.669578658209907 | Down |
| Q5PQZ9 | Ndufc2 | 0.66837219397227 | Down |
| A0A0H2UI06 | Ndufaf6 | 0.666210621595383 | Down |
| P13697 | Me1 | 0.665433300866021 | Down |
| Q5RKI1 | Eif4a2 | 0.66519155104955 | Down |
| F1LZX7 | Rpl17 | 0.664950225088331 | Down |
| F1LR92 | Serpina3m | 0.664742098914253 | Down |
| P16617 | Pgk1 | 0.66400764716996 | Down |
| A0A0G2K0B0 | Cdv3 | 0.66340262360043 | Down |
| Q704E8 | Abcb7 | 0.663145108355416 | Down |
| P47967 | Lgals5 | 0.659443318843842 | Down |
| Q5XHZ0 | Trap1 | 0.658758829037348 | Down |
| Q5RJS6 | Mospd1 | 0.658134172360102 | Down |
| Q5M9G8 | Dcaf11 | 0.658124705155691 | Down |
| Q4G082 | Coq3 | 0.6581113172902 | Down |
| D3ZCI0 | Gk | 0.655874957640965 | Down |
| F1LU71 | Auh | 0.655513905816608 | Down |
| D4A4U3 | Mdp1 | 0.655163404014375 | Down |
| A0A0H2UHS8 | Fxyd1 | 0.653651018937429 | Down |
| Q6IRI3 | Pacsin2 | 0.653261389997271 | Down |
| Q5M8C3 | Serpina4 | 0.652002116044363 | Down |
| P61016 | Pln | 0.651361614465713 | Down |
| E9PT87 | Mylk3 | 0.648178742991553 | Down |
| G3V7K3 | Cp | 0.647374974356757 | Down |
| A0A0G2K7L0 | Mrps10 | 0.646893686718411 | Down |
| D3ZGP9 | Dhrs7c | 0.643153501881493 | Down |
| D3ZIL6 | Echdc2 | 0.641881492402819 | Down |
| Q32PX9 | Afg1l | 0.639960298935572 | Down |
| Q7TP52 | Cmbl | 0.639372630251778 | Down |
| Q8SEZ0 | Mt-nd5 | 0.638955311642753 | Down |
| D4A3D2 | Smyd1 | 0.63527039024565 | Down |
| P09456 | Prkar1a | 0.633054640558031 | Down |
| M0RB63 | LOC684509 | 0.632340848445892 | Down |
| Q9JJW3 | Atp5md | 0.632258693377177 | Down |
| D3ZXA6 | Pdpr | 0.627931675977177 | Down |
| O08836 | Igbp1 | 0.627310726377699 | Down |
| F1M953 | Hspa9 | 0.625938670502769 | Down |
| B1H271 | Slc25a42 | 0.624994827641381 | Down |
| D3Z9R8 | Atp5mpl | 0.62417189280192 | Down |
| Q6AYQ8 | Fahd1 | 0.623990300628874 | Down |
| Q5XIH4 | Lias | 0.623660074339973 | Down |
| Q62920 | Pdlim5 | 0.622747864988115 | Down |
| A0A0G2JSM8 | Cdc42 | 0.622191372844908 | Down |
| P27008 | Parp1 | 0.621573865413666 | Down |
| Q5XI77 | Anxa11 | 0.621495150857502 | Down |
| D4A9G1 | Rpl3l | 0.621148645877838 | Down |
| Q7TP58 | Bpgm | 0.619095557265811 | Down |
| A0A0G2JSH9 | Prdx2 | 0.617453171147241 | Down |
| O35796 | C1qbp | 0.614328225453695 | Down |
| P11348 | Qdpr | 0.609777212142944 | Down |
| Q8SEZ2 | COIII | 0.607345875766542 | Down |
| D3ZS75 | Ndufc1 | 0.605237513780594 | Down |
| Q6AYT0 | Cryz | 0.604655080371433 | Down |
| P07340 | Atp1b1 | 0.60255425175031 | Down |
| A0A0G2JSL7 | Pdp2 | 0.602373232444127 | Down |
| E9PT90 | Spart | 0.601756797896491 | Down |
| F1LRK4 | Grsf1 | 0.601229664352205 | Down |
| D4A478 | Ntpcr | 0.600586632887522 | Down |
| A0A0G2JXH2 | Ccdc51 | 0.600422928730647 | Down |
| Q6WN19 | Rtn2 | 0.599194016721514 | Down |
| Q5FVQ8 | Nlrx1 | 0.597250766224331 | Down |
| D4AB01 | Hint2 | 0.596047706074185 | Down |
| C0HLM6 | Smim20 | 0.594445271624459 | Down |
| A0A0G2JSG6 | Ak2 | 0.593371931049559 | Down |
| Q5XIK2 | Tmx2 | 0.59336305823591 | Down |
| B5DF46 | Pmm2 | 0.593168358008067 | Down |
| A0A0G2QC17 | Pdp1 | 0.58973201447063 | Down |
| F1LQZ0 | Tmem65 | 0.588544057475196 | Down |
| G3V9S6 | Ndufa1 | 0.588230772150888 | Down |
| Q29RW1 | Myh4 | 0.58682212067975 | Down |
| P10818 | Cox6a1 | 0.586339344580968 | Down |
| D3ZK97 | H3f3c | 0.586068820622233 | Down |
| D4A4A9 | Mrpl19 | 0.585888594388962 | Down |
| D2XV59 | Gtpbp1 | 0.585222812162504 | Down |
| G3V9S0 | Cyb5r1 | 0.584733181529575 | Down |
| Q3V5X8 | Endog | 0.58402986990081 | Down |
| A0A0G2JSI1 | Aldh9a1 | 0.58368123571078 | Down |
| Q811X6 | Cryl1 | 0.582497441106372 | Down |
| F7EV94 | Bckdha | 0.580833690034019 | Down |
| D3ZRM9 | LOC100360491 | 0.580481991171837 | Down |
| P97576 | Grpel1 | 0.578123781416151 | Down |
| Q5U329 | Slc4a1 | 0.577967610624101 | Down |
| Q5M7W7 | Pars2 | 0.577935609552595 | Down |
| G3V9T4 | Tmem38a | 0.577107590105798 | Down |
| F1LRV6 | Gmpr | 0.575813763671451 | Down |
| A0A0G2K189 | Scrn3 | 0.575114972061581 | Down |
| D4A1G1 | Acyp2 | 0.574735071923998 | Down |
| P0C5I0 | Cdnf | 0.574454661872652 | Down |
| D4A305 | Ccdc58 | 0.572431266307831 | Down |
| B5DEF6 | RGD1310159 | 0.571814318497976 | Down |
| B2RYW9 | Fahd2 | 0.570070899195141 | Down |
| D3ZH23 | RGD1560917 | 0.56975860397021 | Down |
| G3V879 | Coq7 | 0.569189382923974 | Down |
| Q6AY80 | Nqo2 | 0.568685797353585 | Down |
| Q07803 | Gfm1 | 0.567662947707706 | Down |
| O35244 | Prdx6 | 0.566076685984929 | Down |
| D4AAT4 | Snrpf | 0.566048810879389 | Down |
| D3ZEH6 | Nudt8 | 0.565416521496243 | Down |
| A0JPJ7 | Ola1 | 0.565303925010893 | Down |
| D3ZX74 | Coq10a | 0.564537478817834 | Down |
| A0A0G2K6H2 | Gstz1 | 0.563085102372699 | Down |
| D3ZKG1 | Mmut | 0.562137418323093 | Down |
| P27139 | Ca2 | 0.559968160258399 | Down |
| Q925Q9 | Sh3kbp1 | 0.55938285175297 | Down |
| Q5M7T5 | Serpinc1 | 0.558978249629339 | Down |
| Q5U1W6 | Apool | 0.557925972673628 | Down |
| O88767 | Park7 | 0.556541153126293 | Down |
| F1MAA5 | Rangap1 | 0.55349373486307 | Down |
| G3V8G2 | Psmd5 | 0.55263106028239 | Down |
| Q8HIC6 | Mt-nd4 | 0.552042666408751 | Down |
| P50411 | Ppp1r2 | 0.549858988987075 | Down |
| M0RDD8 | Eml1 | 0.548894461658266 | Down |
| P53534 | Pygb | 0.548187159829669 | Down |
| Q6MGB5 | Hsd17b8 | 0.548053556018405 | Down |
| D3ZZQ4 | Aamdc | 0.547270715236664 | Down |
| D3Z881 | Tbc1d4 | 0.546771412922277 | Down |
| Q4FZT8 | Spryd4 | 0.546739204062356 | Down |
| A0A096MJY1 | Gpc6 | 0.545919573969311 | Down |
| B0K020 | Cisd1 | 0.545502397749159 | Down |
| F1M7Y3 | Rap1gds1 | 0.54535784986284 | Down |
| B2GV75 | D2hgdh | 0.545133024454117 | Down |
| Q6AXR6 | Gsto1 | 0.544841756423315 | Down |
| M0R7G4 | Apoo | 0.544148739841249 | Down |
| Q5M9I2 | Man2c1 | 0.543276710642709 | Down |
| D4A264 | Zadh2 | 0.543233805232578 | Down |
| P97544 | Plpp3 | 0.542540785339143 | Down |
| D3ZEH2 | Foxred1 | 0.542132970359591 | Down |
| A0A0G2K3W1 | Vwa8 | 0.541808038949966 | Down |
| P54001 | P4ha1 | 0.538986117475563 | Down |
| Q5RKI8 | Abcb8 | 0.538226750161912 | Down |
| P12001 | Rpl18 | 0.537495152817832 | Down |
| P97519 | Hmgcl | 0.537225203381644 | Down |
| Q6PCT8 | Sdhd | 0.535922868384255 | Down |
| F1LP30 | Mccc1 | 0.53569080763393 | Down |
| Q2UZS7 | Dnaja3 | 0.535093933343887 | Down |
| A0A096MKG5 | Nadk2 | 0.534962864385711 | Down |
| P62632 | Eef1a2 | 0.533865355783039 | Down |
| Q5I0P2 | Gcsh | 0.532890193992191 | Down |
| A0A0G2JZH8 | Pdhx | 0.532866928312513 | Down |
| P11530 | Dmd | 0.532097793287701 | Down |
| Q4G069 | Rmdn1 | 0.531618171268039 | Down |
| D4AE56 | Ptges2 | 0.531491918696298 | Down |
| Q5XIN6 | Letm1 | 0.528595348199208 | Down |
| P05545 | Serpina3k | 0.526701692905691 | Down |
| Q5XIJ4 | Fam210a | 0.52208470304807 | Down |
| D3ZWS2 | LOC690000 | 0.522064715623856 | Down |
| D3ZUX7 | Acsf3 | 0.520869649118847 | Down |
| B0BMW2 | Hsd17b10 | 0.518947250313229 | Down |
| D3ZXF8 | Mrpl43 | 0.518755485614141 | Down |
| Q9WVJ4 | Synj2bp | 0.514109863175286 | Down |
| Q63704 | Cpt1b | 0.513251430458493 | Down |
| G3V945 | Aldh5a1 | 0.512504481607013 | Down |
| Q9WUS0 | Ak4 | 0.511811157067617 | Down |
| D3ZJS3 | Tomm6 | 0.509103818072213 | Down |
| Q6P790 | Rpl6 | 0.50707931485441 | Down |
| B0BNK6 | Bckdhb | 0.504852271742291 | Down |
| Q68FT8 | Serpinf2 | 0.504537661870321 | Down |
| F1LN92 | Afg3l2 | 0.504090954860052 | Down |
| D3ZA93 | Acot13 | 0.50396031472418 | Down |
| F1LMM8 | Pdk2 | 0.503638454609447 | Down |
| D4A228 | Myom3 | 0.503086859981219 | Down |
| D4AEH9 | Agl | 0.502993156512578 | Down |
| A0A0G2KAQ5 | Myoz2 | 0.502464503049851 | Down |
| D3ZLT1 | Ndufb7 | 0.502209471331702 | Down |
| P16303 | Ces1d | 0.502168973286947 | Down |
| A0A0G2JVG4 | Pecr | 0.501719901959101 | Down |
| D3ZSG3 | Tmod4 | 0.497838391198052 | Down |
| P84817 | Fis1 | 0.497751481003231 | Down |
| M0R515 | Nudt13 | 0.49754289454884 | Down |
| A0A0G2K261 | Iars2 | 0.496300995349884 | Down |
| Q5UAJ5 | ATP8 | 0.495715065134896 | Down |
| D3ZT90 | Gcdh | 0.49373254345523 | Down |
| B2GUZ6 | Rtn4ip1 | 0.492731269862917 | Down |
| A0A0G2K398 | Gpx4 | 0.49265138970481 | Down |
| F1LM33 | Lrpprc | 0.491256472137239 | Down |
| D3ZEV8 | Susd2 | 0.490994524624613 | Down |
| Q58FK9 | Kyat3 | 0.490189396672779 | Down |
| F1LP21 | Timm8a1 | 0.488932347959942 | Down |
| B6DYQ0 | Gstk1 | 0.488846249050564 | Down |
| Q6AY56 | Tuba8 | 0.488400826851527 | Down |
| P97571 | Capn1 | 0.487915204630958 | Down |
| P04041 | Gpx1 | 0.487731799483299 | Down |
| P63031 | Mpc1 | 0.486517445908652 | Down |
| A0A0G2JV20 | Fars2 | 0.485891413357523 | Down |
| Q8SEZ8 | NADH1 | 0.485320281651285 | Down |
| Q920F5 | Mlycd | 0.484222355816099 | Down |
| Q5FVT5 | Pdk1 | 0.483137567838033 | Down |
| G3V8V3 | Pygm | 0.482607728077306 | Down |
| D3ZS58 | Ndufa2 | 0.482281347115835 | Down |
| D4A4P3 | Ndufb3 | 0.48217722442415 | Down |
| B2GV06 | Oxct1 | 0.480525122748481 | Down |
| G3V6H0 | LOC100363782 | 0.479244518611166 | Down |
| F1LY11 | Trdn | 0.47873175309764 | Down |
| P21571 | Atp5pf | 0.477203477587965 | Down |
| Q6AYE2 | Sh3glb1 | 0.476198054022259 | Down |
| B2RYK3 | Spr | 0.472462336222331 | Down |
| M0R5K3 | LOC687508 | 0.471489899688297 | Down |
| Q5XIJ3 | Idh3g | 0.471123486757279 | Down |
| A0A0G2JSV6 | Hba-a2 | 0.471093138058981 | Down |
| Q5XIB3 | Adprhl1 | 0.470399028725094 | Down |
| F1LXA0 | Ndufa12 | 0.464646240075429 | Down |
| Q62651 | Ech1 | 0.463931408193376 | Down |
| S5RKC8 | CYTB | 0.462502499421438 | Down |
| A0A0G2JSR0 | Vdac3 | 0.461779322889116 | Down |
| A0A0G2K1W9 | Ldhd | 0.460095547967487 | Down |
| Q6PDU7 | Atp5mg | 0.459690463211801 | Down |
| G3V8M4 | Cox6a2 | 0.458084389567375 | Down |
| D3ZZN3 | Acss1 | 0.457907034291162 | Down |
| P29419 | Atp5me | 0.457353568739361 | Down |
| D3ZJW6 | rCG_21066 | 0.456897106435564 | Down |
| Q499N5 | Acsf2 | 0.455942645668984 | Down |
| P05426 | Rpl7 | 0.454427955879105 | Down |
| A0A0G2K7K2 | Aifm1 | 0.454366644223531 | Down |
| D3ZUX5 | Chchd3 | 0.453830569982529 | Down |
| D3ZAF6 | Atp5mf | 0.453369935353597 | Down |
| F7F389 | C9 | 0.453020850817362 | Down |
| A0A1B0GWV6 | Aox3 | 0.452600494027138 | Down |
| G3V6L9 | Fkbp3 | 0.451156593031353 | Down |
| A0A0G2JYU2 | mrpl11 | 0.450356385774083 | Down |
| Q7TS56 | Cbr4 | 0.449681401252747 | Down |
| Q4V8I9 | Ugp2 | 0.449267152282927 | Down |
| P07335 | Ckb | 0.449226756890615 | Down |
| P97521 | Slc25a20 | 0.449130972226461 | Down |
| A0A0G2K531 | Gpx3 | 0.448841965860791 | Down |
| B2RYS0 | Cox7a2 | 0.448542478183905 | Down |
| D3Z9I1 | Coa3 | 0.44833223356141 | Down |
| P11980 | Pkm | 0.446859987245666 | Down |
| P17078 | Rpl35 | 0.445545579824183 | Down |
| P29418 | Atp5f1e | 0.444451784094175 | Down |
| A0A0H2UHI5 | Serpina3n | 0.444150257441733 | Down |
| Q68G41 | Eci1 | 0.443856305546231 | Down |
| P29266 | Hibadh | 0.443737324741152 | Down |
| P0DN35 | Ndufb1 | 0.443017625146442 | Down |
| P07943 | Akr1b1 | 0.441096944941415 | Down |
| Q5XIM4 | Dmac2l | 0.439280165566339 | Down |
| Q4G064 | Coq5 | 0.438303560018539 | Down |
| Q5XIE6 | Hibch | 0.43749582933055 | Down |
| P04797 | Gapdh | 0.436984454592069 | Down |
| P48500 | Tpi1 | 0.436868907676803 | Down |
| D4A565 | Ndufb5 | 0.434125542640686 | Down |
| D4A9P9 | Hhatl | 0.433489008082284 | Down |
| Q63910 | Hba-a3 | 0.430779316359096 | Down |
| Q8VID1 | Dhrs4 | 0.43000387152036 | Down |
| Q68FX0 | Idh3B | 0.428521609968609 | Down |
| P29117 | Ppif | 0.42832885020309 | Down |
| Q60587 | Hadhb | 0.428094716535674 | Down |
| Q5XIG1 | Ldb3 | 0.42704956067933 | Down |
| A0A0H2UH99 | Rpl24 | 0.424836244848039 | Down |
| Q68FT4 | Suclg2 | 0.424327327145471 | Down |
| B2RYS8 | Ndufb8 | 0.424066252178616 | Down |
| Q7TQ16 | Uqcrq | 0.423261963658863 | Down |
| M0R959 | Mmab | 0.423024237155914 | Down |
| P36972 | Aprt | 0.422364168696933 | Down |
| M0R629 | Adssl1 | 0.421103987428877 | Down |
| B0BNN3 | Ca1 | 0.42052212688658 | Down |
| F1LRJ9 | Selenbp1 | 0.419778062237634 | Down |
| Q6AYS7 | Acy1a | 0.419122056828605 | Down |
| P12007 | Ivd | 0.416655057006412 | Down |
| G3V640 | Timm44 | 0.415929473108715 | Down |
| O88989 | Mdh1 | 0.413813786374198 | Down |
| D4A7D7 | H6pd | 0.412388996945488 | Down |
| B2RYT5 | Cox7a2l | 0.411882607473267 | Down |
| Q920P0 | Dcxr | 0.409905129008823 | Down |
| P67779 | Phb | 0.409291222691536 | Down |
| Q8SEZ1 | Mt-nd3 | 0.407432956828011 | Down |
| F1M6X5 | Txnrd2 | 0.40734107626809 | Down |
| P97532 | Mpst | 0.406604982084698 | Down |
| G3V7Y3 | Atp5f1d | 0.406144320964813 | Down |
| Q6PCU8 | Ndufv3 | 0.405734760893716 | Down |
| Q5I0K3 | Clybl | 0.404953524470329 | Down |
| Q80W89 | Ndufa11 | 0.404935340086619 | Down |
| A9UMV9 | Ndufa7 | 0.404060492912928 | Down |
| Q6IRS6 | Fetub | 0.401622931162516 | Down |
| A0A0G2K459 | Mtch2 | 0.400933557086521 | Down |
| Q4V8F9 | Hsdl2 | 0.40020258559121 | Down |
| A0A0G2K7F7 | Tpm1 | 0.399899476104313 | Down |
| P05065 | Aldoa | 0.398039586014218 | Down |
| P26772 | Hspe1 | 0.397868012388547 | Down |
| D4A0Y4 | Oxnad1 | 0.397478255960676 | Down |
| Q5XIC0 | Eci2 | 0.395674240258005 | Down |
| Q6PDU6 | Hbb-b1 | 0.395247585243649 | Down |
| A0A0G2JSS8 | Prdx5 | 0.394917026162148 | Down |
| Q80UL1 | Myom1 | 0.394416905111737 | Down |
| D4A830 | Ppa2 | 0.393120808733834 | Down |
| Q5M9I5 | Uqcrh | 0.39029265443484 | Down |
| P38718 | Mpc2 | 0.387537492646112 | Down |
| P14046 | A1i3 | 0.386623288194338 | Down |
| Q64428 | Hadha | 0.383879310554928 | Down |
| F1LZW6 | Slc25a13 | 0.382789388298988 | Down |
| D4A3V2 | Ndufa6 | 0.382328222195308 | Down |
| D3ZCZ9 | LOC100912599 | 0.380840023358663 | Down |
| B5DFN3 | Uqcc2 | 0.379713866445753 | Down |
| C6ZII9 | Sirt3 | 0.379692488246494 | Down |
| P11951 | Cox6c2 | 0.379690231548415 | Down |
| A0A0G2JSH2 | Bdh1 | 0.37833567791515 | Down |
| D3ZZ21 | Ndufb6 | 0.37739708688524 | Down |
| B1H216 | Hba-a1 | 0.377389416098595 | Down |
| Q52KS1 | Pfkm | 0.37737344039811 | Down |
| D3ZVS2 | L2hgdh | 0.377348007427322 | Down |
| P42123 | Ldhb | 0.375920850369665 | Down |
| A0A0H2UI21 | Crat | 0.372169219785267 | Down |
| B2RYS2 | Uqcrb | 0.37194237112999 | Down |
| B0BNE6 | Ndufs8 | 0.369722548458311 | Down |
| A0A0G2JSV0 | Ndrg2 | 0.36880326933331 | Down |
| B2RZD6 | Ndufa4 | 0.368721678853035 | Down |
| Q5XIH7 | Phb2 | 0.362959264053239 | Down |
| Q5XIC2 | Ecsit | 0.358982318805324 | Down |
| Q5RK08 | Nipsnap2 | 0.358050091399087 | Down |
| P19511 | Atp5pb | 0.356675134764777 | Down |
| Q5XIF3 | Ndufs4 | 0.355322102705638 | Down |
| B5DEL8 | Ndufs5 | 0.353305627902349 | Down |
| G3V6H5 | Slc25a11 | 0.352579449613889 | Down |
| D4A7L4 | Ndufb11 | 0.350396977530585 | Down |
| Q9Z311 | Mecr | 0.349682827790578 | Down |
| P14408 | Fh | 0.34919684794214 | Down |
| P08461 | Dlat | 0.348369393911626 | Down |
| P10860 | Glud1 | 0.344582610660129 | Down |
| P08503 | Acadm | 0.34330952167511 | Down |
| A0A0G2K5P5 | Myom1 | 0.342999388774236 | Down |
| Q63362 | Ndufa5 | 0.342687441243066 | Down |
| M0R7S5 | Plin4 | 0.342582199308608 | Down |
| A0A0G2JVL6 | Ndufa8 | 0.341679197218683 | Down |
| D3ZYU4 | Cox20 | 0.34075320760409 | Down |
| P15429 | Eno3 | 0.340693599647946 | Down |
| Q2TA68 | Opa1 | 0.339034785827001 | Down |
| G3V7I0 | Prdx3 | 0.337368475066291 | Down |
| B4F768 | Aldh4a1 | 0.332518973284298 | Down |
| Q5XIG9 | Mtfp1 | 0.332034176008569 | Down |
| A0A0G2JWS2 | Nebl | 0.331275641918182 | Down |
| P20788 | Uqcrfs1 | 0.3312140090598 | Down |
| P08009 | Gstm7 | 0.33120280255874 | Down |
| B2RYW3 | Ndufb9 | 0.32929537528091 | Down |
| Q6UPE1 | Etfdh | 0.328351828787062 | Down |
| F1LNF7 | Idh3a | 0.327796694305208 | Down |
| Q5XIT9 | Mccc2 | 0.326409553488096 | Down |
| P31399 | Atp5pd | 0.324567741817898 | Down |
| P24329 | Tst | 0.323839942614238 | Down |
| B2GV15 | Dbt | 0.323717436856694 | Down |
| P15650 | Acadl | 0.322116807103157 | Down |
| P10888 | Cox4i1 | 0.321646531422933 | Down |
| G3V936 | Cs | 0.318485870957375 | Down |
| P17764 | Acat1 | 0.317485705018043 | Down |
| D3ZF13 | Ndufab1 | 0.316844585869048 | Down |
| A0A0G2K5F1 | Macrod1 | 0.31664298971494 | Down |
| A0A0G2JVH4 | Immt | 0.316401806142595 | Down |
| F1LPG5 | Ndufb4 | 0.314866516325209 | Down |
| A0A482IDN3 | Hspd1 | 0.312322119871775 | Down |
| D3ZG43 | Ndufs3 | 0.309615279237429 | Down |
| Q9QVC8 | Fkbp4 | 0.30896789001094 | Down |
| Q6AYQ4 | Tmem109 | 0.30791700962517 | Down |
| Q6AXV4 | Samm50 | 0.302462688750691 | Down |
| Q5U2X7 | Timm21 | 0.302021854453617 | Down |
| Q6PCU0 | Atp5f1c | 0.300188953677813 | Down |
| Q4PP99 | Tnnc1 | 0.299384421772427 | Down |
| D4A0T0 | Ndufb10 | 0.296914029452536 | Down |
| Q6P9Y4 | Slc25a4 | 0.29604061279032 | Down |
| Q6IRH6 | Slc25a3 | 0.294962439272139 | Down |
| P00507 | Got2 | 0.292540518773927 | Down |
| P39069 | Ak1 | 0.290248190363248 | Down |
| P21913 | Sdhb | 0.290118785368072 | Down |
| P12075 | Cox5b | 0.289325974053807 | Down |
| G3V6P2 | Dlst | 0.289084209336175 | Down |
| P13803 | Etfa | 0.287585573063956 | Down |
| P23693 | Tnni3 | 0.287058545483483 | Down |
| Q920L2 | Sdha | 0.282976393898328 | Down |
| P32551 | Uqcrc2 | 0.282967841459645 | Down |
| Q641Y2 | Ndufs2 | 0.28060494363308 | Down |
| Q5BK63 | Ndufa9 | 0.278064744340049 | Down |
| Q06647 | Atp5po | 0.276775426334805 | Down |
| P19234 | Ndufv2 | 0.275373460517989 | Down |
| Q561S0 | Ndufa10 | 0.274605244398117 | Down |
| D3ZE15 | Ndufa13 | 0.272481019298236 | Down |
| Q68FU3 | Etfb | 0.271979855166541 | Down |
| Q6P6R2 | Dld | 0.270428818960985 | Down |
| Q01177 | Plg | 0.269872988263766 | Down |
| P52873 | Pc | 0.268786799576547 | Down |
| A0A0G2KAM3 | Pdhb | 0.26843467189206 | Down |
| G3V7J0 | Aldh6a1 | 0.267410066392687 | Down |
| Q5BJQ0 | Coq8a | 0.2652804205815 | Down |
| Q66HF1 | Ndufs1 | 0.264852687716484 | Down |
| Q5XIH3 | Ndufv1 | 0.26139069100221 | Down |
| F1LX07 | Slc25a12 | 0.261382434103224 | Down |
| F1LM47 | Sucla2 | 0.26062857442432 | Down |
| P11507 | Atp2a2 | 0.260421545141273 | Down |
| Q5RJN0 | Ndufs7 | 0.259845341245333 | Down |
| G3V9R9 | Afm | 0.254821195370621 | Down |
| P07895 | Sod2 | 0.254371979170376 | Down |
| Q68FY0 | Uqcrc1 | 0.252009044090907 | Down |
| Q9Z2L0 | Vdac1 | 0.244847690065702 | Down |
| P11240 | Cox5a | 0.240457963612344 | Down |
| P13221 | Got1 | 0.240230820245213 | Down |
| D3ZY44 | Mrps2 | 0.240095807446374 | Down |
| P14604 | Echs1 | 0.239610720011923 | Down |
| Q6IMX3 | Acads | 0.236112099554804 | Down |
| F1LQ95 | Tnnt2 | 0.23347249130408 | Down |
| P81155 | Vdac2 | 0.232133054071003 | Down |
| P04166 | Cyb5b | 0.230459532803959 | Down |
| O35115 | Fhl2 | 0.230098240905338 | Down |
| Q5M9H2 | Acadvl | 0.227571863267157 | Down |
| P85834 | Tufm | 0.227099656230874 | Down |
| Q4FZZ4 | Pdha1 | 0.226602340737978 | Down |
| P18886 | Cpt2 | 0.222972601652145 | Down |
| A0A0H2UHE1 | Suclg1 | 0.221653075681792 | Down |
| G3V7K1 | Myom2 | 0.219663244154718 | Down |
| Q68FT1 | Coq9 | 0.219001821345753 | Down |
| G3V8U8 | Bcat2 | 0.218451109197405 | Down |
| Q06QH5 | ND2 | 0.216375450500184 | Down |
| D3ZD09 | Cox6b1 | 0.213054589099354 | Down |
| P20761 | Igh-1a | 0.212982252240181 | Down |
| P16290 | Pgam2 | 0.207809704873297 | Down |
| G3V9U2 | Acaa2 | 0.205473483436638 | Down |
| D3ZGK7 | Ces1c | 0.199947165118323 | Down |
| B6RK61 | Myh7b | 0.199363462626934 | Down |
| P18163 | Acsl1 | 0.196925967931747 | Down |
| P07483 | Fabp3 | 0.192917983565066 | Down |
| Q5XI78 | Ogdh | 0.191197079088953 | Down |
| D3ZFQ8 | Cyc1 | 0.18159084684319 | Down |
| S5RZM8 | COX2 | 0.181374478671286 | Down |
| Q9WVK7 | Hadh | 0.18111484663354 | Down |
| A0A0G2JZ73 | Serpina1 | 0.178090087241597 | Down |
| Q5M7V3 | LOC367586 | 0.177895386392872 | Down |
| Q68FY4 | Gc | 0.172895219590929 | Down |
| B0LPN4 | Ryr2 | 0.166871411104997 | Down |
| P24090 | Ahsg | 0.154128472010295 | Down |
| P20059 | Hpx | 0.15206439461973 | Down |
| P02767 | Ttr | 0.114976464460293 | Down |
| Q7TMB9 | LOC299282 | 0.0957520773841274 | Down |
| A0A0G2JSH5 | Alb | 0.0883021569914289 | Down |

|  |  |  |  |
| --- | --- | --- | --- |

**Table S2** A total of 154 overlapping proteins in DCM

| Gene | Protein ID | DCM:Ctrl | Regulation |
| --- | --- | --- | --- |
| Nrap | D3Z802 | 5.96535136964586 | UP |
| Fhl1 | Q6P792 | 4.7820783191257 | UP |
| Pdlim3 | A0A0G2JSM3 | 4.12320196628571 | UP |
| Xirp1 | D4ABA9 | 4.05154222912259 | UP |
| Flnc | A0A0H2UHR7 | 4.02099582884047 | UP |
| Stat3 | P52631 | 3.8580293920305 | UP |
| Pdk4 | G3V778 | 3.5478585296207 | UP |
| Apob | F1M6Z1 | 3.28613670667013 | UP |
| Dsg2 | F1LYX9 | 3.20354607370165 | UP |
| Csrp3 | G3V7U0 | 3.12674122386509 | UP |
| Cryab | P23928 | 3.06163342793783 | UP |
| Jup | Q6P0K8 | 3.04002888997396 | UP |
| Actn1 | Q6GMN8 | 2.97337714831034 | UP |
| Tuba1A | P68370 | 2.93094746271769 | UP |
| Rtn4 | F1LQN3 | 2.8961593442493 | UP |
| Myh11 | E9PTU4 | 2.8175534274843 | UP |
| Hspb7 | B5DFG4 | 2.74747241867913 | UP |
| Vwf | F1M957 | 2.74631817473306 | UP |
| Vcl | R9PXU6 | 2.72133677535587 | UP |
| Dsp | F1LMV6 | 2.42628524038527 | UP |
| Cltc | F1M779 | 2.39287430710263 | UP |
| Bgn | P47853 | 2.37684945265452 | UP |
| Rras2 | Q5BJU0 | 2.25802822907766 | UP |
| Ankrd1 | Q8R560 | 2.24781941043006 | UP |
| Scp2 | F1LQ55 | 2.16177230411106 | UP |
| Actn4 | A0A0G2K013 | 2.14843099647098 | UP |
| Tpm3 | Q63610 | 2.11106989118788 | UP |
| Myh10 | G3V9Y1 | 2.0521185596784 | UP |
| Lmna | G3V8L3 | 2.04164588451385 | UP |
| Ctnnb1 | A0A0G2JT93 | 2.03893955548604 | UP |
| Itgb1 | A0A0G2JSK5 | 2.02367566691505 | UP |
| Cdh2 | Q9Z1Y3 | 2.01377432876163 | UP |
| Apoa1 | P04639 | 18.6980391608344 | UP |
| Postn | A0A097BW25 | 11.0645656585693 | UP |
| Flna | C0JPT7 | 1.96078198485904 | UP |
| Hspb6 | P97541 | 1.91857843928867 | UP |
| Obscn | A0A0G2JUP3 | 1.88143086433411 | UP |
| Fbln5 | Q9WVH8 | 1.88118678993649 | UP |
| Ilk | Q99J82 | 1.86136489444309 | UP |
| Mapk3 | P21708 | 1.83357090420193 | UP |
| Rmnd1 | Q1W176 | 1.82970456944571 | UP |
| Dnajc30 | B2RYV0 | 1.73703791035546 | UP |
| Calr | P18418 | 1.70014446311527 | UP |
| Ttn | Q5VJM4 | 1.67061428891288 | UP |
| Myl6 | Q64119 | 1.62750089168548 | UP |
| Ctsd | Q6P6T6 | 1.62605779700809 | UP |
| Lmo7 | Q6JBI7 | 1.61281866497464 | UP |
| Myzap | Q5EB94 | 1.60599055555132 | UP |
| Ahcy | P10760 | 1.5966186788347 | UP |
| Pam16 | D3ZZV1 | 1.59651279449463 | UP |
| Sgcg | Q5XID6 | 1.58612877792782 | UP |
| Eif4H | Q5XI72 | 1.56866447793113 | UP |
| Myh2 | G3V6E1 | 1.55496921804216 | UP |
| Cav3 | P51638 | 1.54828655719757 | UP |
| Rac1 | Q6RUV5 | 1.53872195217345 | UP |
| F2 | G3V843 | 1.53140766090817 | UP |
| Cap2 | P52481 | 1.52991131941478 | UP |
| Hspa4 | F1LRV4 | 1.52659728129705 | UP |
| Ldb3 | A0A096MJ01 | 1.50324022769928 | UP |
| Tfam | Q91ZW1 | 0.669578658209907 | Down |
| Pln | P61016 | 0.651361614465713 | Down |
| Cp | G3V7K3 | 0.647374974356757 | Down |
| Mt-Nd5 | Q8SEZ0 | 0.638955311642753 | Down |
| Pdlim5 | Q62920 | 0.622747864988115 | Down |
| Rpl3L | D4A9G1 | 0.621148645877838 | Down |
| Pmm2 | B5DF46 | 0.593168358008067 | Down |
| Ndufa1 | G3V9S6 | 0.588230772150888 | Down |
| Myh4 | Q29RW1 | 0.58682212067975 | Down |
| Gfm1 | Q07803 | 0.567662947707706 | Down |
| Mt-Nd4 | Q8HIC6 | 0.552042666408751 | Down |
| D2Hgdh | B2GV75 | 0.545133024454117 | Down |
| Foxred1 | D3ZEH2 | 0.542132970359591 | Down |
| Hmgcl | P97519 | 0.537225203381644 | Down |
| Sdhd | Q6PCT8 | 0.535922868384255 | Down |
| Eef1A2 | P62632 | 0.533865355783039 | Down |
| Dmd | P11530 | 0.532097793287701 | Down |
| Bckdhb | B0BNK6 | 0.504852271742291 | Down |
| Myom3 | D4A228 | 0.503086859981219 | Down |
| Myoz2 | A0A0G2KAQ5 | 0.502464503049851 | Down |
| Fis1 | P84817 | 0.497751481003231 | Down |
| Gcdh | D3ZT90 | 0.49373254345523 | Down |
| Mlycd | Q920F5 | 0.484222355816099 | Down |
| Trdn | F1LY11 | 0.47873175309764 | Down |
| Cox6A2 | G3V8M4 | 0.458084389567375 | Down |
| Slc25A20 | P97521 | 0.449130972226461 | Down |
| Coa3 | D3Z9I1 | 0.44833223356141 | Down |
| Atp5F1E | P29418 | 0.444451784094175 | Down |
| Coq5 | Q4G064 | 0.438303560018539 | Down |
| Gapdh | P04797 | 0.436984454592069 | Down |
| Tpi1 | P48500 | 0.436868907676803 | Down |
| Ppif | P29117 | 0.42832885020309 | Down |
| Hadhb | Q60587 | 0.428094716535674 | Down |
| Ndufb8 | B2RYS8 | 0.424066252178616 | Down |
| Mdh1 | O88989 | 0.413813786374198 | Down |
| Mt-Nd3 | Q8SEZ1 | 0.407432956828011 | Down |
| Txnrd2 | F1M6X5 | 0.40734107626809 | Down |
| Atp5F1D | G3V7Y3 | 0.406144320964813 | Down |
| Tpm1 | A0A0G2K7F7 | 0.399899476104313 | Down |
| Myom1 | Q80UL1 | 0.394416905111737 | Down |
| Ppa2 | D4A830 | 0.393120808733834 | Down |
| Hadha | Q64428 | 0.383879310554928 | Down |
| Ndufa6 | D4A3V2 | 0.382328222195308 | Down |
| Sirt3 | C6ZII9 | 0.379692488246494 | Down |
| Ndufs8 | B0BNE6 | 0.369722548458311 | Down |
| Ndufs4 | Q5XIF3 | 0.355322102705638 | Down |
| Ndufb11 | D4A7L4 | 0.350396977530585 | Down |
| Acadm | P08503 | 0.34330952167511 | Down |
| Opa1 | Q2TA68 | 0.339034785827001 | Down |
| Nebl | A0A0G2JWS2 | 0.331275641918182 | Down |
| Etfdh | Q6UPE1 | 0.328351828787062 | Down |
| Acadl | P15650 | 0.322116807103157 | Down |
| Cox4I1 | P10888 | 0.321646531422933 | Down |
| Cs | G3V936 | 0.318485870957375 | Down |
| Macrod1 | A0A0G2K5F1 | 0.31664298971494 | Down |
| Hspd1 | A0A482IDN3 | 0.312322119871775 | Down |
| Tnnc1 | Q4PP99 | 0.299384421772427 | Down |
| Slc25A4 | Q6P9Y4 | 0.29604061279032 | Down |
| Slc25A3 | Q6IRH6 | 0.294962439272139 | Down |
| Sdhb | P21913 | 0.290118785368072 | Down |
| Etfa | P13803 | 0.287585573063956 | Down |
| Tnni3 | P23693 | 0.287058545483483 | Down |
| Sdha | Q920L2 | 0.282976393898328 | Down |
| Uqcrc2 | P32551 | 0.282967841459645 | Down |
| Ndufs2 | Q641Y2 | 0.28060494363308 | Down |
| Ndufa9 | Q5BK63 | 0.278064744340049 | Down |
| Ndufv2 | P19234 | 0.275373460517989 | Down |
| Etfb | Q68FU3 | 0.271979855166541 | Down |
| Dld | Q6P6R2 | 0.270428818960985 | Down |
| Ndufs1 | Q66HF1 | 0.264852687716484 | Down |
| Ndufv1 | Q5XIH3 | 0.26139069100221 | Down |
| Atp2A2 | P11507 | 0.260421545141273 | Down |
| Sod2 | P07895 | 0.254371979170376 | Down |
| Vdac1 | Q9Z2L0 | 0.244847690065702 | Down |
| Cox5A | P11240 | 0.240457963612344 | Down |
| Acads | Q6IMX3 | 0.236112099554804 | Down |
| Tnnt2 | F1LQ95 | 0.23347249130408 | Down |
| Fhl2 | O35115 | 0.230098240905338 | Down |
| Acadvl | Q5M9H2 | 0.227571863267157 | Down |
| Pdha1 | Q4FZZ4 | 0.226602340737978 | Down |
| Cpt2 | P18886 | 0.222972601652145 | Down |
| Myom2 | G3V7K1 | 0.219663244154718 | Down |
| Coq9 | Q68FT1 | 0.219001821345753 | Down |
| Cox6B1 | D3ZD09 | 0.213054589099354 | Down |
| Myh7B | B6RK61 | 0.199363462626934 | Down |
| Fabp3 | P07483 | 0.192917983565066 | Down |
| Hadh | Q9WVK7 | 0.18111484663354 | Down |
| Ryr2 | B0LPN4 | 0.166871411104997 | Down |
| Ttr | P02767 | 0.114976464460293 | Down |
| Alb | A0A0G2JSH5 | 0.0883021569914289 | Down |
