# Supplementary figures and images for: Insight into the underlying molecular mechanism of dilated cardiomyopathy through integrative analysis of data mining, iTRAQ-PRM proteomics and bioinformatics
Source: Proteome Sci. 2023 Sep 22;21:13. doi: 10.1186/s12953-023-00214-9 (PMC10517512; doi:10.1186/s12953-023-00214-9)

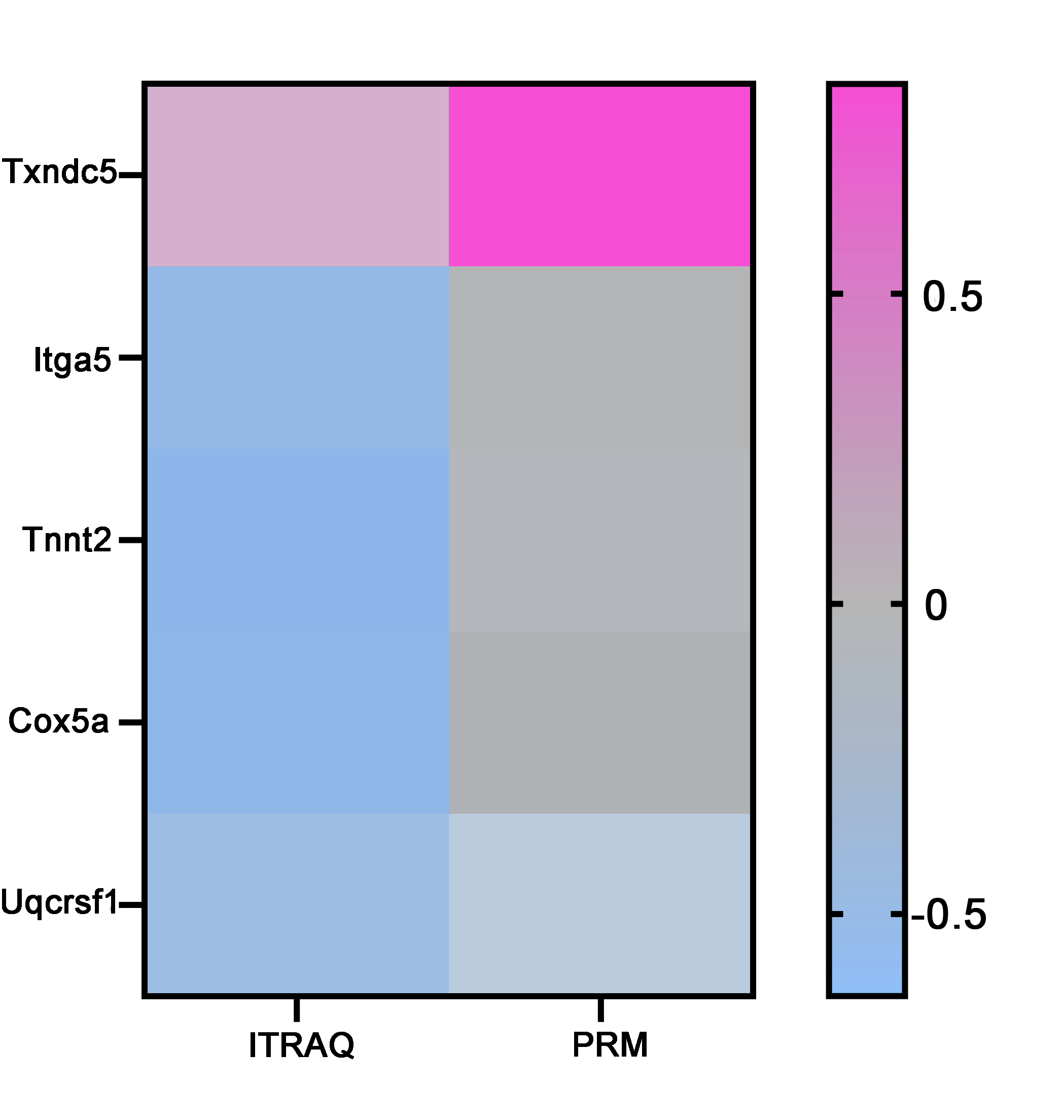

Supplement: Supplementary file 2 — Additional file 2: Figure S1. PRM workflow: the skyline platform supports PRM-based targeted MS Quantification. [file 12953_2023_214_MOESM2_ESM.tif]
